# CHK1 inhibitor induced PARylation by targeting PARG causes excessive replication and metabolic stress and overcomes chemoresistance in ovarian cancer

**DOI:** 10.1038/s41420-024-02040-0

**Published:** 2024-06-11

**Authors:** Ganesh Acharya, Chinnadurai Mani, Naresh Sah, Karunakar Saamarthy, Robert Young, Mark B. Reedy, Robert W. Sobol, Komaraiah Palle

**Affiliations:** 1 Department of Cell Biology and Biochemistry, School of Medicine, Texas Tech University Health Sciences Center, Lubbock, TX USA; 2 Department of Obstetrics & Gynecology, School of Medicine, Texas Tech University Health Sciences Center, Lubbock, TX USA; 3 Department of Pathology and Laboratory Medicine, Warren Alpert Medical School, & Legorreta Cancer Center, Brown University, Providence, RI USA

**Keywords:** Cancer metabolism, Cancer therapeutic resistance

## Abstract

Chemoresistance contributes to the majority of deaths in women with ovarian cancer (OC). Altered DNA repair and metabolic signaling is implicated in mediating therapeutic resistance. DNA damage checkpoint kinase 1 (CHK1) integrates cell cycle and DNA repair in replicating cells, and its inhibition causes replication stress, repair deficiency and cell cycle dysregulation. We observed elevated Poly-ADP-ribosylation (PAR) of proteins (PARylation) and subsequent decrease in cellular NAD^+^ levels in OC cells treated with the CHK1 inhibitor prexasertib, indicating activation of NAD^+^ dependent DNA repair enzymes poly-ADP-ribose polymerases (PARP1/2). While multiple PARP inhibitors are in clinical use in treating OC, tumor resistance to these drugs is highly imminent. We reasoned that inhibition of dePARylation by targeting Poly (ADP-ribose) glycohydrolase (PARG) would disrupt metabolic and DNA repair crosstalk to overcome chemoresistance. Although PARG inhibition (PARGi) trapped PARylation of the proteins and activated CHK1, it did not cause any significant OC cell death. However, OC cells deficient in CHK1 were hypersensitive to PARGi, suggesting a role for metabolic and DNA repair crosstalk in protection of OC cells. Correspondingly, OC cells treated with a combination of CHK1 and PARG inhibitors exhibited excessive replication stress-mediated DNA lesions, cell cycle dysregulation, and mitotic catastrophe compared to individual drugs. Interestingly, increased PARylation observed in combination treatment resulted in depletion of NAD^+^ levels. These decreased NAD^+^ levels were also paralleled with reduced aldehyde dehydrogenase (ALDH) activity, which requires NAD^+^ to maintain cancer stem cells. Furthermore, prexasertib and PARGi combinations exhibited synergistic cell death in OC cells, including an isogenic chemoresistant cell line and 3D organoid models of primary patient-derived OC cell lines. Collectively, our data highlight a novel crosstalk between metabolism and DNA repair involving replication stress and NAD^+^-dependent PARylation, and suggest a novel combination therapy of CHK1 and PARG inhibitors to overcome chemoresistance in OC.

## Introduction

Ovarian cancer (OC) is the most lethal gynecological malignancy and leading causes of cancer-related deaths in women in the United States and globally [1] (https://seer.cancer.gov/statistics/). This is primarily due to its insidious progression that masks early detection and thus OC is diagnosed commonly at the disease stage III or IV [2]. Despite their initial responses to frontline platinum-taxane-based chemotherapeutic regimens, majority of the patients develop resistance and relapse [3, 4]. These relapsed or recurrent tumors are more aggressive, resistant to available chemotherapeutic drugs and contributes to most of the OC-related deaths [4–6]. Recently, multiple poly (ADP-ribose) polymerase (PARP) inhibitors (PARPi) were approved to treat patients with germline mutations in *BRCA1/2* and associated genes that cause deficiency in repair of DNA double-strand breaks through homologous recombination (HR) [7]. These PARP-targeted therapies show significant therapeutic benefits in OC patients with HR deficiency through a synthetic lethality mechanism [7–9]. However, over 50% of the OC patients who are DNA repair proficient do not benefit from PARPi [10, 11]. Additionally, recent clinical data shows that large number of patients with *BRCA* mutations do not respond well to the PARPi [10, 12]. Even the patients who respond initially also develop resistance to PARP-targeted therapeutics eventually [6, 11–13]. This clinical need underscores the necessity for novel therapeutic strategies and paradigms to treat and overcome chemoresistance in OC.

DNA damage response (DDR) and repair genes, including *PARP1* are upregulated in OC to promote tumor cell survival from the genotoxic lesions caused by metabolic and replication stress and in response to DNA damage therapy [14–16]. PARP1 is one of the early response proteins that senses both DNA single and double-strand breaks, and stalled replication forks [17]. Upon sensing damaged DNA, PARP1 utilizes NAD^+^ to synthesize poly-ADP-ribose (PAR) and rapidly catalyzes post-translational modification (PARylation) of itself, neighboring histones, and other proteins [18]. These act as protein scaffold and facilitates timely recruitment of additional DDR and repair factors to the sites of damaged DNA to complete repair [19]. In cells, PARylation is a transient and dynamic process, timely removal of PAR or dePARylation is critical from the proteins for efficient repair of DNA lesions and cells’ recovery from genotoxic stress [20, 21]. Since PARP enzymes use NAD^+^ as the substrate to synthesize PAR, cellular NAD^+^ levels play a critical role in regulation of PARP1 activity [22]. Thus, reduced NAD^+^ levels inhibit PARylation of proteins and repair complex formation and impair cells’ ability to repair damaged DNA [23]. Moreover, unrestricted PARylation also causes imbalance in cellular homeostasis due to excessive depletion of NAD^+^ levels, which can lead to cell death [24, 25]. Thus, both PARylation and de-PARylation processes have been a target for development of cancer therapeutics.

Poly(ADP-ribose) glycohydrolase (PARG) is a major cellular enzyme responsible for rapid de-PARylation of PARP synthesized PAR and generates monomeric ADP-ribose units [18, 26]. Similar to PARP proteins, PARG is also recruited to the sites of damaged DNA and removes excessive PARylation to prevent cell death [27]. PARG deficiency impairs cells’ ability to repair both DNA single and double-strand breaks [23]. Thus, PARG inhibitors are shown to exhibit antitumor activity against several cancer cells including those from ovarian, breast, pancreatic, lung, and brain cancers [15, 23, 28–31]. Recent studies showed PARG inhibitor sensitizes OC cells to platinum drugs and PARP-targeted therapies [12, 32, 33]. Interestingly, genetic screens in OC cell line panels identified synthetic lethality of replication factors with PARGi, and inhibition of DNA damage checkpoint kinase 1 (CHK1) in combination with PARGi caused synergistic lethality [15]. Nevertheless, the precise mechanistic details underlying the impact of PARGi on CHK1 activation, cell cycle progression, and OC survival remain elusive. Conversely, the involvement of PARG in response to CHK1 inhibition has not been thoroughly investigated, especially concerning PARylation and de-PARylation processes, and its potential role in overcoming OC chemoresistance. Recent studies have shed light on the divergent responses of OC cell lines to PARGi, indicating opportunities for strategic combinations that enhance replication catastrophe in these model systems [32].

Dysregulated DNA replication and cell division, driven by oncogenes and growth factors results in metabolic and replication stress, which may cause DNA damage and cell death [34, 35]. CHK1 is a critical regulator of replication progression and facilitates timely repair of replication stress-associated DNA damage by activating S and G2 checkpoints [36]. Inhibition of CHK1 in tumor cells causes enhanced replication stress and mitotic catastrophe [37, 38]. Additionally, we and others showed that CHK1 regulates integrity of the Fanconi anemia-BRCA-RAD51 repair complex and HR-mediated repair of DSB and sensitizes tumor cells to PARP-targeted therapies [37–40]. Further, we have found that PARGi leads to elevated CHK1 activation in glioma stem cells and cell lines, along with enhanced apoptosis [23, 31]. These studies suggest an important role for PARP and PARG in response to CHK1 inhibition and repair of DNA lesions and cell survival. While resistance to PARPi is inevitable, we reasoned targeting PARG would trap PARylated proteins on the DNA, inhibits efficient DNA repair and recovery from replication stress and cause cellular imbalance in NAD^+^ levels and causes cell death. Additionally, it was not known whether inhibiting PARG will reduce the availability of cellular NAD^+^ due to DNA damage-induced heavy PARylation and over-utilization of NAD^+^. Moreover, NAD^+^ is an important factor for the aldehyde dehydrogenase enzymes, and to maintain OC stem cells [41]. Therefore, we reasoned that inhibition of CHK1 causes increased replication stress due to stalled/collapsed replication forks and HR deficiency, and prolonged activation of PARP enzymes and PARylation of proteins. In these conditions, inhibition of PARG could lead to NAD^+^ imbalance and effectively kill OC stem cells and overcome chemoresistance.

We propose a novel synthetic lethal approach, combining prexasertib with the PARG inhibitor *PDD00017273* to counteract chemoresistance in ovarian cancer (OC). Our hypothesis is rooted in the idea that inhibiting PARG can enhance the replication stress induced by CHK1 inhibitors. This occurs by capturing PARylated proteins on chromatin, compromising DNA repair mechanisms, and culminating in cancer cell death by inducing metabolic and mitotic catastrophes.

## Results

### CHK1 inhibition causes increased PARylation of proteins in OC cells

**Fig. 1:**
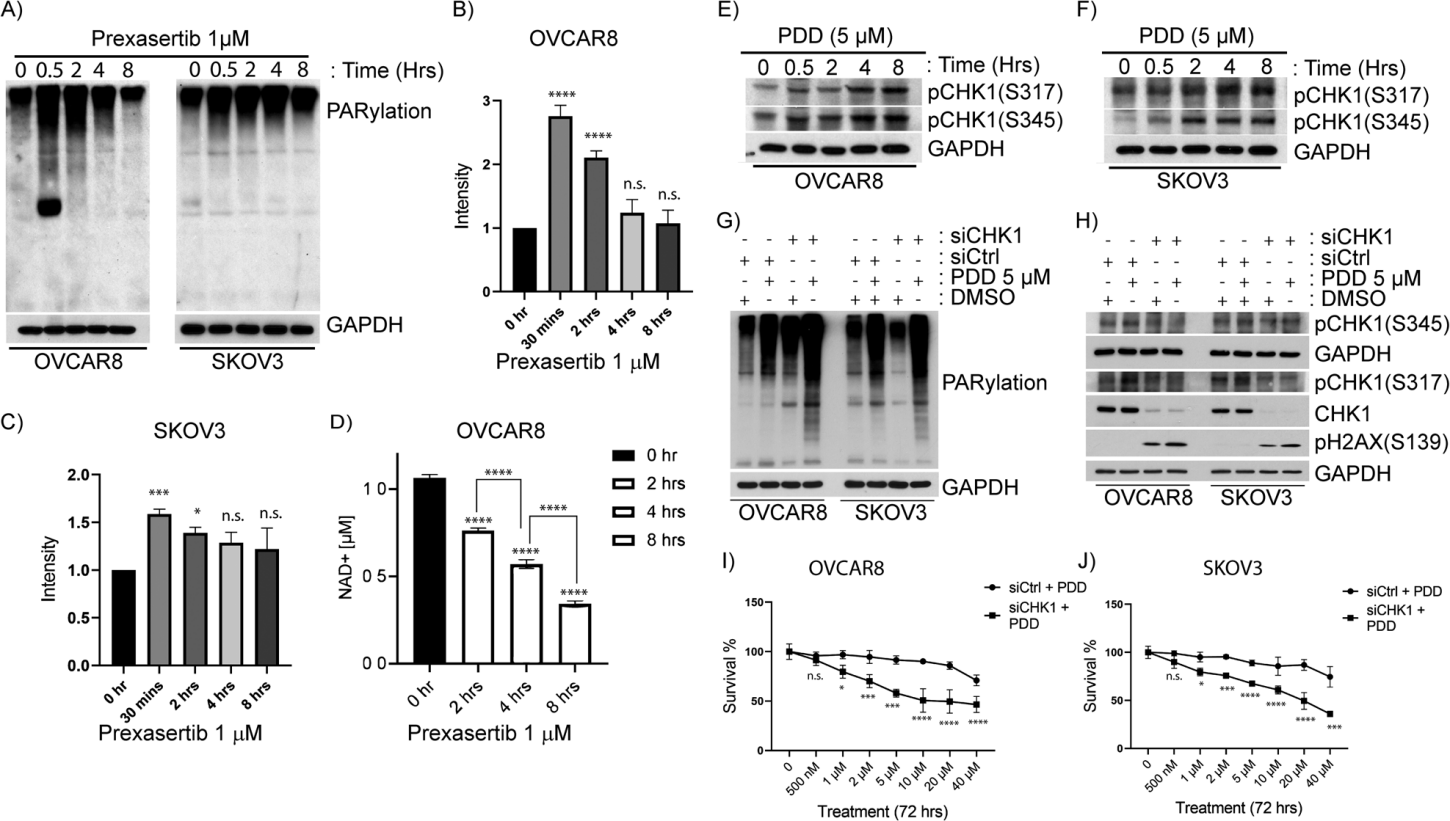
CHK1 inhibition activates PARP and induces PARylation of proteins and replication stress-induced CHK1 phosphorylation in OC cells. **A** Immunoblot analysis shows PARP1 mediated/activated PARylation of proteins induced by the treatment of CHK1 inhibitor, prexasertib at 1 µM concentration in OC cells- OVCAR8 and SKOV3 cells in time dependent manner. **B**, **C** Quantification of elevated levels of PARylated proteins induced by the treatment of 1 µM prexasertib in OVCAR8 and SKOV3 cells, respectively. ImageJ was used to quantify the intensity of the proteins. **D** NAD^+^ assay shows reduced levels of cellular NAD^+^ upon prexasertib treatment at 1 µM concentration in OVCAR8 cells. Immunoblot analysis depicting activation of ATR-mediated phosphorylation of CHK1 at S317 and S345 in OC cells **E** OVACR8 and **F** SKOV3 cells after treatment with 5 µM of PARG inhibitor*, PDD00017273* for 24 h. **G** Immunoblot analysis showing downregulation of CHK1 along with 5 µM of PDD treatment for 24 h showed increased levels of PARylation of proteins and similarly, **H** increased levels of a DNA damage marker-pH2AX(S139) in OC cells. **I**, **J** Survival curve showing downregulation of CHK1 along with 5 µM of PDD treatment for 24 h showed decreased survival percentage of cells in OVCAR8 and SKOV3 cells, respectively. All the experiments were performed in triplicates and the bar graph denotes their standard deviation. Two-way ANOVA using Bonferroni’s multiple comparison tests were performed to analyze the statistical significance. n.s. not significant; *p < 0.05; ***p < 0.001; ****p < 0.0001.

CHK1 inhibition causes replication stress and HR-mediated DNA repair deficiency (HRD) in *BRCA*-proficient ovarian, breast, and other cancer cells [37, 42–45]. PARP1/2-mediated PARylation and subsequent dePARylation by PARG regulates repair of DNA lesions and cancer cell survival. To examine the influence of CHK1 inhibition on PARylation of proteins, we exposed OC cell lines OVCAR8 and SKOV3 to 1 µM prexasertib and analyzed PARylation of proteins at different exposure times ranging from 30 min to 8 h. As shown in Fig. 1A, prexasertib treatment caused increased PARylation of proteins in both OVCAR8 and SKOV3 cells compared to their respective untreated controls. Interestingly, within 30 min, prexasertib induced maximum increase in PARylation of proteins in both cell lines. However, longer exposure to prexasertib (4 and 8 h) did not cause any further increase in global PARylated proteins. Indeed, increased PARylation of proteins were not significant at the longer time exposures (4 and 8 h) compared to their respective controls (Fig. 1B, C). Although the pattern of protein PARylation in OVCAR8 and SKOV3 cell lines varies, the overall intensity of PARylated proteins were significantly increased to their respective untreated controls. On the other hand, when OC cells were treated with nanomolar concentrations (1–40 nM) of prexasertib, increased PARylation was observed even after 24 h exposure (Supplementary Fig. 1A–C). The variations in PARylation patterns can be ascribed to dynamic shifts in PARylation and dePARylation, governed by PARP and PARG enzymes, respectively. The decline in cellular NAD^+^ levels following prexasertib exposure suggests ongoing PARP-mediated PARylation in these cells (Fig. 1D). Conversely, PARG-mediated dePARylation of proteins may contribute to maintaining balance and preventing cell death.

### PARGi in OC cells induces replication stress and ATR-mediated CHK1 phosphorylation

PARG is the major cellular enzyme that exhibits exo and endo-glycohydrolase activity and responsible for recycling ADP-ribose [46]. To examine whether inhibition of PARG in OC cells activates CHK1, we treated OVCAR8 and SKOV3 cells with PARG inhibitor (*PDD00017273*, abbreviated as PDD) and evaluated activation of CHK1 by measuring its phosphorylation. PARGi activated ATR-mediated phosphorylation of CHK1 at S317 and S345 in OVCAR8 and SKOV3 cells as shown in Fig. 1E, F, respectively. Phosphorylation of CHK1 was increased with the PDD exposure time. However, PDD treatment alone did not cause any significant differences in cell cycle profiles compared to control cells (Supplementary Fig. 2A, B). This indicates the mild replication stress caused by PARGi could be timely fixed by intact ATR/CHK1 mediated DDR and repair. Consistent with this, treatment of OC cells with PDD alone did not cause significant cytotoxicity to OC cells at lower concentrations. On the other hand, knocking down CHK1 in these cells showed increased sensitivity to PARGi as evidenced by increased levels of PARylation of proteins (Fig. 1G), increased pH2AX(S139) (Fig. 1H), and decreased survival percentage of cells in OVCAR8 (Fig. 1I) and SKOV3 cells (Fig. 1J).

Collectively, these findings indicate that CHK1 plays a protective role against replication stress induced by PARG inhibition. On the other hand, inhibition of CHK1 results in replication stress-induced DNA lesions and the PARylation of proteins. This necessitates the involvement of PARG for the resolution of PAR, and to facilitate the repair of damaged DNA and cell survival. Therefore, combination of CHK1 inhibition and PARG inhibition can cause intense replication stress, deficiency in DNA damage repair, cell cycle dysregulation, and synergistic OC cancer cell death.

### PDD in combination with prexasertib causes cell cycle dysregulation, increased DNA damage and changes in nuclear morphology

**Fig. 2:**
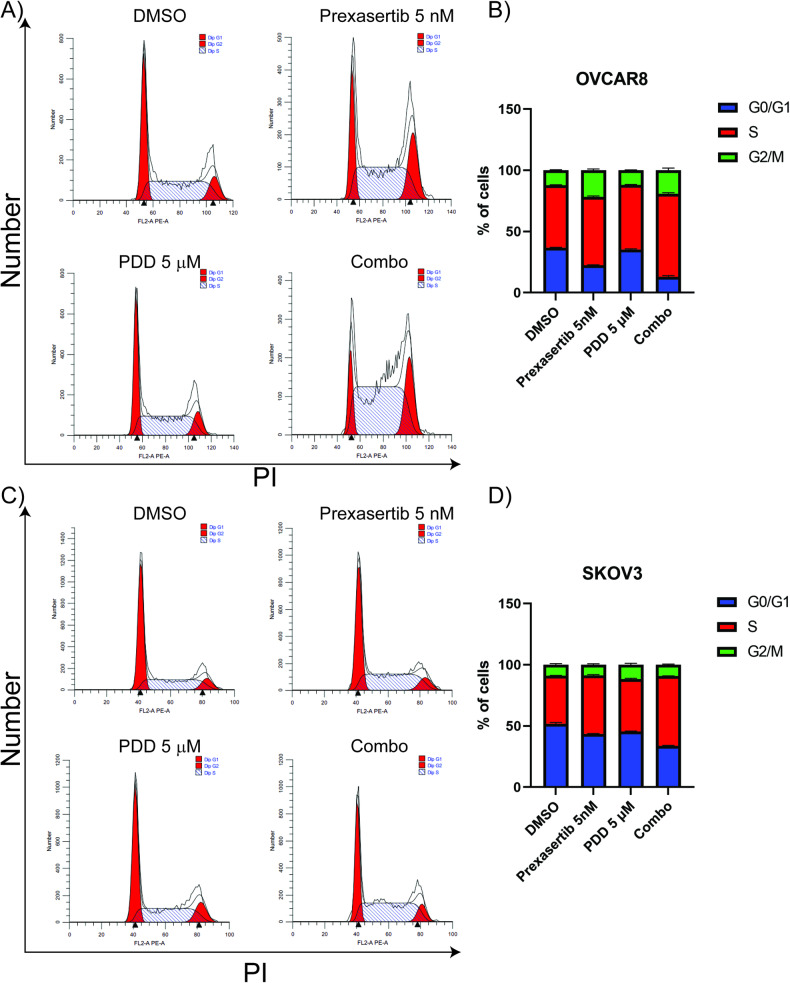
PDD in combination with prexasertib causes cell cycle dysregulation in OC cells. **A**, **C** Cell cycle profile of OC cells treated with 5 nM prexasertib, 5 µM PDD and their combination for 24 h in OVCAR8 and SKOV3 cells, respectively. **B**, **D** Histogram representation of cell cycle profile in OC cells treated with 5 nM prexasertib, 5 µM PDD and their combination for 24 h in OVCAR8 and SKOV3 cells, respectively. Error bars represent standard deviation from three independent experiments.

Previous studies from our lab and others have shown that prexasertib treatment induces replication stress, cell cycle dysregulation and mitotic catastrophe in breast, ovarian, and other cancer cell lines [37, 38, 47, 48]. Consistently, prexasertib treatment caused increased accumulation of cells in S and G2 phases of the cell cycle in OVCAR8 cells (Supplementary Fig. 3A, B). As described previously, inhibition of PARG alone did not cause any notable changes in the cell cycle profiles of SKOV3 cells (Supplementary Fig. 2A, B). Nevertheless, PDD treatment impelled prexasertib-induced dysregulation of cell cycle in both OVCAR8 and SKOV3 cells. This was clearly evidenced by the substantial increase in accumulation of cells in the S-phase, as compared to the effects observed with individual drug treatments (Fig. 2A–D).

**Fig. 3:**
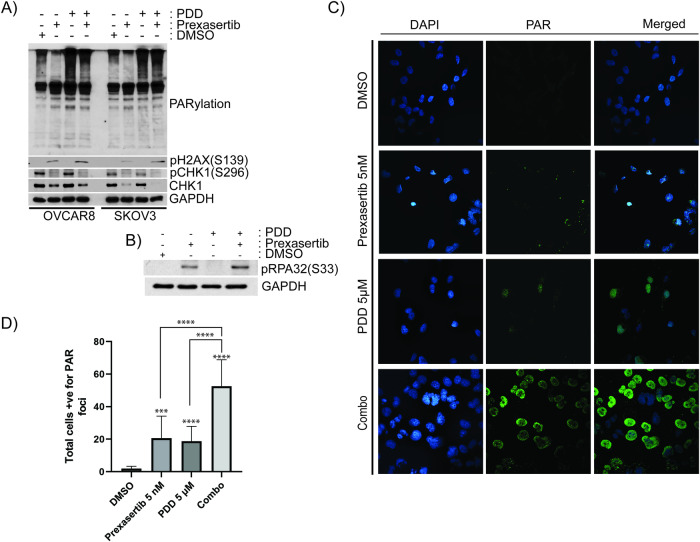
PDD traps prexasertib-induced PARylated proteins on chromatin. **A** Immunoblot analysis showing PARylation of proteins, pH2AX(S139), pCHK1(S296), and CHK1 basally as well as after treatment with 5 nM prexasertib, 5 µM PDD and their combination in OC cells. **B** Immunoblotting showing pRPA32(S33) in SKOV3 cells treated with DMSO, 5 nM prexasertib, 5 µM PDD and their combination. **C** Immunofluorescence study showing PAR foci staining in SKOV3 cells treated with DMSO, 5 nM prexasertib, 5 µM PDD and their combination. **D** Histogram representation of PAR foci staining in SKOV3 cells treated with DMSO, 5 nM prexasertib, 5 µM PDD and their combination. 50 cells were counted for each group for an experiment, and the data presented are an average of 3 different experiments. All the experiments were repeated three times, and the bar graph denotes their standard deviation. One-way ANOVA using Dunnett’s T3 multiple comparison tests were performed to analyze the statistical significance. ***p < 0.001; ****p < 0.0001.

To gain further mechanistic insights on combination of CHK1 and PARG inhibition, OC cells were treated with prexasertib and PDD as single agents as well as combination treatments and examined total PARylation of proteins and replication stress markers. As shown in both immunoblotting (Fig. 3A, B) and immunofluorescence studies (Fig. 3C, D), inhibition of either CHK1 or PARG caused increased levels of PAR staining compared to vehicle-treated cells. Interestingly, cells treated in combination with both CHK1 and PARG inhibitors showed significantly more elevated levels of PAR staining compared to individual drugs. These results indicate that CHK1 inhibition caused increased DNA lesions, which in turn induced PARP1-mediated PARylation of proteins. In these conditions, PARG inhibition caused trapping of PARylated protein on chromatin resulting in elevated PARylation.

**Fig. 4:**
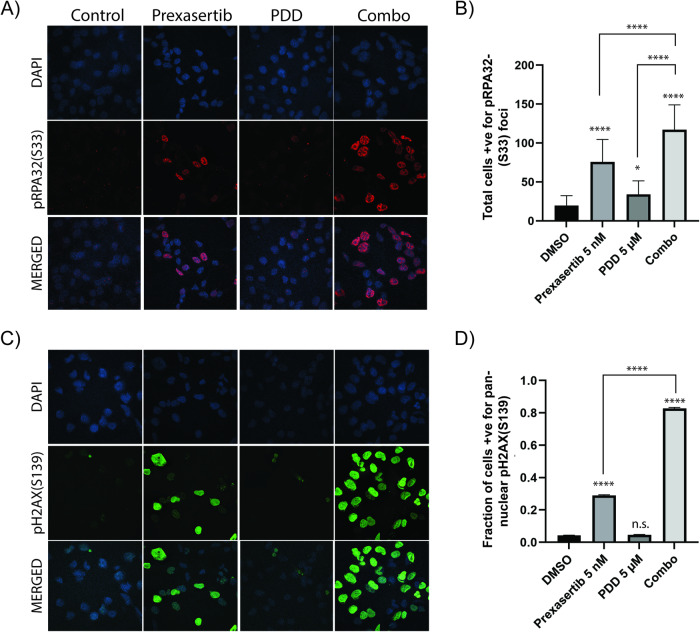
Combination of prexasertib and PDD treatment causes replication stress and DNA damage. **A** Immunofluorescence study showing pRPA32(S33) foci in SKOV3 cells treated with DMSO, 5 nM prexasertib, 5 µM PDD and their combination. **B** Histogram representation of total cells positive for pRPA32(S33) foci in SKOV3 cells treated with DMSO, 5 nM prexasertib, 5 µM PDD and their combination. A total of 50 cells from each of the three different experiments were counted for our histogram. **C** Immunofluorescence study showing pH2AX(S139) foci in SKOV3 cells treated with DMSO, 5 nM prexasertib, 5 µM PDD and their combination. **D** Histogram representation of percentage of cells with total pan-nuclear pH2AX(S139) staining in SKOV3 cells treated with DMSO, 5 nM prexasertib, 5 µM PDD and their combination. A total of 50 cells from each of the three different experiments were counted for our histogram. Error bars represent the mean ± standard deviation in case of pRPA32(S33) foci and mean ± standard error of mean (SEM) in case of pH2AX(S139). One-way ANOVA using Tukey’s multiple comparison tests were performed to analyze the statistical significance. n.s. not significant; *p < 0.05; ****p < 0.0001.

Consistent with previous studies, prexasertib-treated cells showed inhibition of CHK1 in its autophosphorylation at Ser-296 and induced replication stress as indicated by phosphorylation of pH2AX(S139) and pRPA32(S33) in both immunoblots (Fig. 3A, B). These results were further confirmed by immunofluorescent studies as indicated by pRPA32(S33) foci (Fig. 4A, B) and pan-nuclear staining of pH2AX(S139) (Fig. 4C, D). Although PDD treatment caused increased levels of pRPA32(S33) foci compared to vehicle-treated cells, they were significantly low compared to prexasertib-treated cells. However, combination of prexasertib and PDD treatment caused significantly elevated pRPA32(S33) foci (Fig. 4A, B) as well as protein levels (Fig. 3B) compared to individual drugs.

Moreover, these stalled or unresolved replication fork intermediates can lead to increased replication stress-mediated DNA lesions. To examine whether these cells show increased collapsed forks, we first evaluated pH2AX(S139), a marker for DSB and stalled or collapsed replication forks in these cells. Immunoblot data showed increased levels of pH2AX(S139) in OVCAR8 and SKOV3 cells in comparison to vehicle control cells and prexasertib or PDD as individual drugs (Fig. 3A). Similar with pRPA32(S33) foci data, the cells treated with prexasertib and PDD combination showed significantly elevated levels of pH2AX(S139) foci compared to vehicle-treated and individual drug treated SKOV3 cells (Fig. 4C, D).

**Fig. 5:**
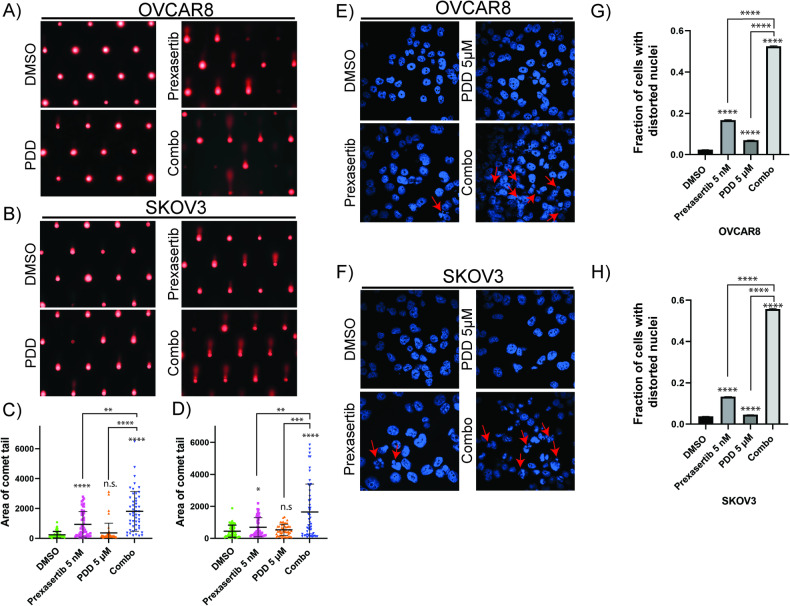
PDD sensitizes prexasertib-induced DNA damage and increases nuclear distortion. **A**, **B** Comet assay representative images treated with DMSO, 5 nM prexasertib, 5 µM PDD and their combination for 24 h in OVCAR8 and SKOV3, respectively. **C**, **D** Analysis of comet tail area in more than 50 cells from three independent experiments with their standard deviation as the error bars in OVCAR8 and SKOV3, respectively. **E**, **F** Distorted nuclei representative images in DAPI-stained nucleus of OVCAR8 and SKOV3, respectively treated with DMSO, 5 nM prexasertib, 5 µM PDD and their combination for 24 h. **G**, **H** Percentage of cells with distorted nuclei analyzed more than 200 cells from three different experiments with their standard error of mean as the error bars. One-way ANOVA using Dunnett’s T3 multiple comparison test for comet assay and Tukey’s multiple comparison tests for nuclear distortion assay were performed to analyze the statistical significance. n.s. not significant; *p < 0.05; **p < 0.01; ***p < 0.001; ****p < 0.0001.

To further confirm these drug combinations caused increased chromosomal DNA lesions, we performed alkaline COMET-assays to measure both single and double-strand breaks. As shown in the figure, nuclei of OVCAR8 (Fig. 5A, C) and SKOV3 (Fig. 5B, D) cells treated with DMSO showed minimal COMET tail DNA. Consistent with the pH2AX(S139) foci data, prexasertib treated cells showed increased levels of COMET tail DNA compared to vehicle-treated and PDD-treated cells. Although PDD-treated cells increased levels of COMET tail DNA compared to vehicle-treated cells, it was not significantly high. As expected, cells treated with prexasertib and PARGi combination showed several folds increase in levels of COMET tail DNA respective to their individual treatments.

CHK1 inhibition abrogates intra-S and G2 checkpoints, and as a result, DNA synthesis continues with unrepaired DNA lesions and prematurely enters into mitosis, which leads to mitotic catastrophe, characterized by a loss of nuclear membrane integrity and fragmented nuclear morphology. Especially, morphological changes such as distorted nuclei results in mitotic catastrophe, which arises from uneven distribution of chromosomes or chromosome fragments between daughter nuclei due to inadequate separation during cytokinesis [37, 49]. To determine whether these drug combination treatments caused fragmented or distorted nuclei-related morphological changes, we examined the structure and morphology of OC cell nuclei. While prexasertib treatment alone increased nuclear distortion compared to vehicle only treated control, the prexasertib + PDD combination increased distorted nuclei by threefold to fourfold in both the OC cell lines in comparison to prexasertib treatment alone (Fig. 5E–H). Despite the increased presence of distorted nuclei, which could be due to damaged chromosomes either arising from uneven distribution of chromosomes or chromosome fragments between daughter nuclei, a possible indicator of mitotic catastrophe. It is noteworthy that cells subjected to PDD treatment also exhibited a 1.2-fold increase compared to the control group. This could be due to unresolved dePARylation due to PARG inhibition. Collectively, these results indicate CHK1 inhibition-induced DNA lesions persuade PARP1-mediated PARylation of proteins, and in these conditions, inhibition of PARG further traps these PARylated proteins leading to inhibition of DNA repair and recovery from replication stress.

To further validate this hypothesis, OVCAR8 cells were treated with 100 nM of prexasertib for 2 h, washed and released into growth medium with or without 5 µM of PDD for 24 h and analyzed the cell cycle progression by flow cytometry. Interestingly, transient prexasertib treatment caused significant delay in cell cycle progression compared to untreated cells. These cells accumulated more in late S and G2 phases. On the other hand, PDD-treated cells did not show any significant changes in the cell cycle profile compared to vehicle treated cells. However, the cells released into PDD after transient prexasertib treatment were mostly in S phase and did not progress towards G2 phase accumulated more in S phase (Supplementary Fig. 4A–E). Together, these results further support that PARGi causes accumulation of PARylated proteins on the chromatin and inhibits DNA repair and recovery from replication stress caused by prexasertib. Furthermore, these results also suggest an effective combination therapy involving CHK1 and PARG inhibitors for OC.

### CHK1 inhibition in combination with PARGi causes synergistic lethality in OC cell lines

**Fig. 6:**
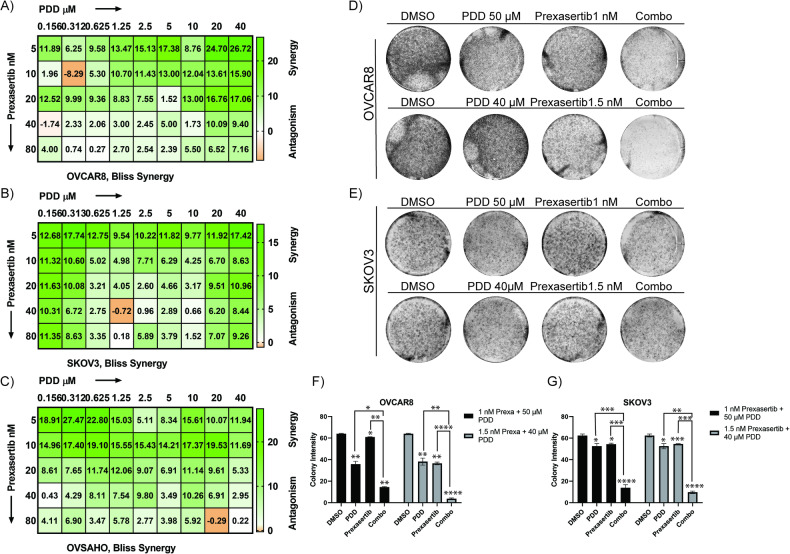
Prexasertib in combination with PDD causes synergistic lethality in OC cells. **A**–**C** Representative Bliss synergy plots of indicated OC cell lines concurrently treated with serial dilutions of prexasertib, PDD and prexasertib plus PDD in OVCAR8, SKOV3 and OVSAHO, respectively. **D**, **E** Colony assay plate wells of OC cells treated with DMSO, 1 nM prexasertib, 50 µM PDD and their combination as well as DMSO, 1.5 nM prexasertib, 40 µM PDD and their combination in both OVCAR8 and SKOV3, respectively. **F**, **G** Histogram representation of colony intensity in OC cells treated with two different concentrations of both prexasertib and PDD as well as their combinations in OVCAR8 and SKOV3, respectively. Error bars represent the standard deviation from three independent experiments in both OC cells. Two-way ANOVA with Tukey’s multiple comparison tests were performed to analyze the statistical significance. n.s. not significant; *p < 0.05; **p < 0.01; ***p < 0.001; ****p < 0.0001.

To evaluate the combination of CHK1 and PARG inhibitors on OC cells, we performed PrestoBlue^TM^ cytotoxicity assays and used a Bliss model to calculate the effects of drugs combinations as reported previously [50, 51]. In this model, the value of 1 is additive and the corresponding positive and negative values indicate synergistic and antagonistic effects respectively, at the indicated concentrations of the drugs combination. As shown in the BLISS drug combination interactions charts in Fig. 6A–C, over 90% of the prexasertib and PDD combinations are synergistic in OVCAR8, and SKOV3 and OVSAHO cell lines, respectively. To further confirm the efficacy of prexasertib and PDD combination in OC cells, we performed high-density clonogenic survival assays. We selected two different concentrations of prexasertib (1 and 1.5 nM), and five different concentrations of PDD (5, 10, 20, 40, and 50 µM) in OVCAR8 and (1, 2, 5, 10, 20 µM) in SKOV3 cells that are less than their IC50 values (Table 1). These concentrations were deliberately set below the IC50 value for each respective drug. Our objective was to assess the efficacy of drug combinations rather than focusing solely on individual treatments. Both OVCAR8 (Supplementary Fig. 5A, D) and SKOV3 (Supplementary Fig. 5E, H) cells showed decrease in colony intensity with increasing concentrations of PDD.

**Table 1:** IC50 value for Prexasertib and PDD in SKOV3 and OVCAR8 cells.

	Prexasertib (nM) (IC50 ± SE)	PDD (µM) (IC50 ± SE)
SKOV3	8.752 ± 1.458	79.75 ± 8.782
OVCAR8	3.259 ± 0.152	193.8 ± 31.30

PrestoBlue cell viability assay was performed to measure the IC50.

Consistent with the BLISS model data, when these cells were treated in combination with 1 and 1.5 nM of prexasertib, significant decrease in colony intensity were observed compared to individual drugs in both OVCAR8 (Supplementary Fig. 5B–D) and SKOV3 (Supplementary Fig. 5F–H) cells. In addition to high-density clonogenic survival assay, we also performed low density clonogenic survival assay and counted the number of colonies to confirm the efficacy of prexasertib and PDD combination in OVCAR8 and SKOV3 cells. We selected two different concentrations of prexasertib (1 nM and 1.5 nM), and two different concentrations of PDD (5 µM and 10 µM). Both OVCAR8 (Supplementary Fig. 6A, C) and SKOV3 (Supplementary Fig. 6B, D) cells showed decrease in colony count in the drug combinations compared to their respective individual treatments.

We further selected two different prexasertib concentrations and two PDD concentrations that were less than the IC_50_ values for the individual drugs and evaluated the effects of the combined drugs at each of these concentrations relative to the single drugs. Our results showed significantly decreased colony intensity in both OVCAR8 (Fig. 6D, F) and SKOV3 (Fig. 6E, G) cells for the combination treatment relative to prexasertib and PDD individual drugs treatment. We analyzed these data using COMPUSYN software and calculated combination index values. As shown in Table 2 (OVCAR8) and Table 3 (SKOV3), most of the drug combinations are either additive or synergistic in both the cell lines.

**Table 2:** Combination index calculation in OVCAR8 cells with PDD and prexasertib.

Dose prexasertib (nM)	Dose PDD (µM)	Effect	CI	Dose prexasertib (nM)	Dose PDD (µM)	Effect	CI
1	5	0.81	1.01543	1.5	5	0.47	0.95007
1	10	0.69	1.05061	1.5	10	0.3	0.85457
1	20	0.47	0.93181	1.5	20	0.18	0.78582
1	40	0.33	0.96398	1.5	40	0.094	0.71282
1	50	0.23	0.84719	1.5	50	0.06	0.64405

**Table 3:** Combination index calculation in SKOV3 cells with PDD and prexasertib.

Dose prexasertib (nM)	Dose PDD (µM)	Effect	CI	Dose prexasertib (nM)	Dose PDD (µM)	Effect	CI
1	1	0.8	0.21627	1.5	1	0.71	0.09205
1	2	0.72	0.18005	1.5	2	0.53	0.06172
1	5	0.54	0.16154	1.5	5	0.41	0.08404
1	10	0.33	0.10949	1.5	10	0.25	0.06737
1	20	0.22	0.10945	1.5	20	0.15	0.06108

### Prexasertib and PDD combination showed synergy in platinum drug resistance cell line and in 3D organoids model of primary patient-derived ovarian tumor cells

**Fig. 7:**
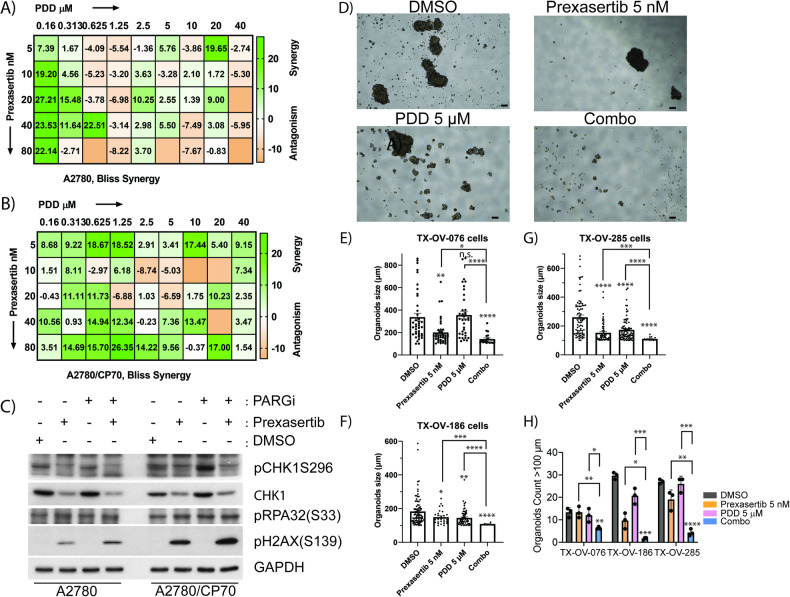
Prexasertib and PDD combination showed synergy in platinum drug resistance cell line as well as 3D organoids models of patient-derived ovarian tumor cells. **A**, **B** Representative Bliss synergy plots of indicated OC cell lines concurrently treated with serial dilutions of prexasertib, PDD and prexasertib plus PDD in A2780 cells and its isogenic platinum resistant model A2780/CP70, respectively. **C** Immunoblot study showing activation of DNA damage protein pH2AX(S139), activation of replication stress marker pRPA32(S33) and phosphorylation of DNA damage response maker CHK11 at S317 in both A2780 cells and its isogenic platinum resistant model A2780/CP70 cells. **D** Representative OC patient-derived primary 3D tumor organoids (TX-OV-285 cells) treated with DMSO, 5 nM prexasertib, 5 µM PDD and their combination. Scale bar represents 100 µm. **E**–**G** Histogram representative of organoids sizes more than 100 µm treated with DMSO, 5 nM prexasertib, 5 µM PDD and their combination for 7-10 days in TX-OV-076, TX-OV-186, and TX-OV-285 cells, respectively. **H** Histogram representative of number of organoids with size more than 100 µm treated with DMSO, 5 nM prexasertib, 5 µM PDD and their combination for 7–10 days in TX-OV-076, TX-OV-186, and TX-OV-285 cells. The organoids size (length) of >100 µm from fifteen images taken from three independent experiments were quantified and error bars represent the standard error of mean. One-way ANOVA with Games-Howell’s multiple comparison tests were performed to analyze statistical significance. n.s. not significant; *p < 0.05; **p < 0.01; ***p < 0.001; ****p < 0.0001.

Chemoresistance and disease recurrence are the major clinical problems that contribute to the majority of OC deaths. To examine whether CHK1 and PARG inhibitor combination effectively kill chemoresistant OC cells, we performed PrestoBlue^TM^ cytotoxicity assays in isogenic platinum-sensitive A2780 and platinum-resistant A2780/CP70 cells and analyzed the Bliss Synergy score. Cisplatin resistance in the A2780/CP70 cell line was 13-fold higher than in A2780 cells [52]. As shown in the BLISS drug combination score chart in Fig. 7A, the parent chemosensitive A2780 cell line showed synergy only at fewer drug combinations that are mostly at lower drug concentrations. At higher drugs concentrations, the combination effects are either additive or antagonistic in these cells. Since this cell line is sensitive to most of the drugs, the single drug treatments alone may cause enough cytotoxicity, and thus suggests combination of drugs may not be needed. Importantly, the combination of prexasertib and PDD showed excellent synergy in platinum-resistant A2780/CP70 cells in most of the combinations of drug concentrations used, indicating the effectiveness of CHK1 and PARG inhibitor combination in killing chemoresistant OC cells (Fig. 7B). To further examine any differences in replication stress-mediated DDR in these cell lines, we evaluated the levels of CHK1 activation and pH2AX(S139). Interestingly, PDD did not cause any changes in detectable levels of pCHK1(S296), in chemosensitive A2780 cells, either as single agent or in combination with prexasertib. Conversely, platinum-resistant A2780/CP70 cells showed increased pCHK1(S296) in response to same concentrations of PDD (Fig. 7C). Additionally, prexasertib as single treatment and in combination with PDD caused increased levels of pH2AX(S139) in A2780/CP70 cells compared to parent A2780 cells. These results indicate elevated levels of DNA damage responses in chemoresistant A2780/CP70 cell line compared to isogenic sensitive A2780 cells may explain the increased synergistic effects of CHK1 and PARG inhibitors drug combination in these cells.

Patient-derived primary tumor organoids are better representative of OC biology and pathophysiological environment and better restore the traits of tumor heterogeneity compared to established immortalized cancer cell lines [53]. To validate the effectiveness of prexasertib + PDD combination, we tested in three different (TX-OV-076, TX-OV-186, and TX-OV-285) OC patient-derived primary 3D tumor organoids (TOs) cultures. As shown in Fig. 7D–G all three TOs showed reduction in number of TOs and their size to both prexasertib and PDD individual drug treatments. Remarkably, prexasertib and PDD combination significantly reduced the organoids size and number (Fig. 7H) in all three TO models compared to individual drugs.

### PARG inhibition augments prexasertib-induced DNA damage, imbalance in cellular NAD^+^ and affects aldehyde dehydrogenase activity, and reduces OC cell stemness

**Fig. 8:**
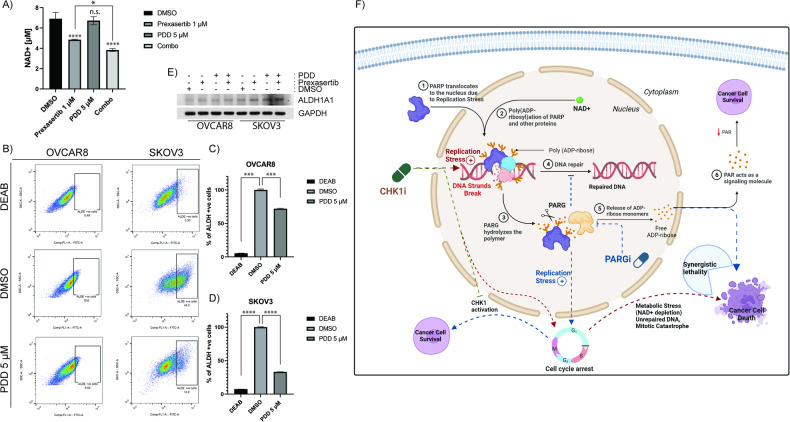
PDD treatment induces prexasertib-induced NAD^+^ depletion and reduces cancer cell stemness in OC cells. **A** NAD^+^ assay shows PDD treatment together with 1 µM prexasertib showed more reduced levels of cellular NAD^+^ compared to prexasertib individual treatment in OVCAR8 cells. **B** Represeantative AldeFluor assay shows depleted ALDH positive cells while treated 5 µM PDD compared to DMSO treated control in OC cells. DEAB was used here is an ALDH negative control. **C**, **D** Histogram representation of percentage of ALDH-positive cells treated with DEAB, DMSO, and 5 µM PDD in OVCAR8 and SKOV3, respectively. **E** Immunoblot study showing the protein expression level of ALDH1A1 while OC cells were treated with DMSO, 5 nM prexasertib, 5 µM PDD, and their combination. All the experiments were done in triplicates and the error bars represent the standard deviation from three independent experiments. **F** Hypothetical/working model illustrating the synergetic lethality with CHK1 and PARG inhibition in ovarian cancer (OC) cells. One-way ANOVA with Tukey’s multiple comparison test for NAD^+^ assay and Dunnett’s T3 multiple comparison test for AldeFluor assay were performed to analyze the statistical significance. n.s. not significant; *p < 0.05; ***p < 0.001; ****p < 0.0001.

DNA damage induced PARylation of proteins expends cellular NAD^+^, which in turn causes metabolic stress as shown in Fig. 1D. We speculated further inhibition of PARG in these conditions prevents hydrolysis of PAR and it recycles and creates excessive metabolic stress in the cells. This was confirmed by NAD^+^ assay as significantly more NAD^+^ was depleted with CHK1 inhibition together with PARG inhibition compared to individual drugs treatment in OVCAR8 cells (Fig. 8A).

Cancer stem cells (CSCs) are the major contributors to chemoresistance in different cancers including OC and represent a novel therapeutic target for OC treatment [54]. It has been already established that aldehyde dehydrogenase isoform I (ALDH1A1) is a stemness marker in HGSOC [55]. As PARP enzymes uses NAD^+^ to synthesize PAR, ALDHs also utilizes NAD^+^ as a redox cofactor for their catalytic activity [56]. Therefore, we hypothesized that reduced NAD^+^ due to inhibition of PARG and CHK1 could potentially affect ALDH1A1 activity and might play an important role in reducing CSCs properties in OC cells. We performed ALDEFLUOR assays to evaluate ALDH activity using flow cytometry. Intriguingly, inhibition of PARG by 5 µM PDD significantly depleted ALDH1A1 activity in both OVCAR8 (Fig. 8B, C) and SKOV3 (Fig. 8B, D) cells. However, ALDH1A1 protein levels were not affected by PARG inhibition (Fig. 8E). These data indicate additional therapeutic and mechanistic insights of PARG inhibition on eliminating CSCs by affecting Aldehyde dehydrogenase activity. This also supports 3D organoid growth inhibitory activity of PDD, a characteristic of CSCs in primary patient-derived OC cell lines (Fig. 7D–H). However, this needs further careful evaluation.

## Discussion

Several CHK1 inhibitors were developed as anticancer drugs and were evaluated for their efficacy either as monotherapy or in combination with other ‘standard of care’ drugs [37, 38, 45, 48, 57, 58]. The majority of these clinical trials were halted, including prexasertib due to toxicities caused by these drugs [59, 60]. CHK1 is a critical player in the ATR-mediated replication stress (RS) induced checkpoint responses and facilitates replication fork stability and timely repair of DNA double-strand breaks through error-free homologous recombination (HR) [36]. We and others have previously shown that CHK1 inhibition causes HRD in *BRCA*-proficient breast, ovarian and other cancer cells, and sensitizes cancer cells to PARP-targeted therapies [37, 38, 61–63]. In this study, CHK1 inhibitor prexasertib caused intense PARylation of proteins in OC cells, within 30 min of exposure. Decreased cellular NAD^+^ levels indicate rapid activation of PARP1/2 enzymes, as they utilize NAD^+^ to synthesize ADP ribose and PARylation. However, PARylation is a transient process and dePARylation of proteins is important for efficient repair of DNA lesions and the cell’s recovery from replication stress and cell cycle progression [15]. Consistently, inhibition of dePARylation by PARGi attenuated recovery of cells from prexasertib-induced replication stress and caused delay in cell cycle progression.

PARG is one of the major cellular enzyme responsible for resolving dePARylation of proteins and recycling of PAR [64]. Thus, our study shows that inhibition of PARG in cancer cells causes an imbalance in ADP-ribose and cellular NAD^+^ homeostasis leading to metabolic and replication stress. Although PARGi alone caused accumulation of PARylation and ATR-CHK1 DNA damage responses, we did not observe any significant changes in cell cycle profile and cytotoxic effects to OC cells. These results suggest that the mild replication stress caused due to accumulation of PARylation in PARGi cells could have been resolved by intact CHK1. This is also evident that CHK1 deficient OC cells show increased sensitivity to PARGi, when compared to CHK1 proficient cells. However, a careful analysis of PARP1 and PARG activities and cellular NAD^+^ levels are important to gain further mechanistic insights.

In agreement with previous studies, PARGi causes synergistic lethality when combined with DNA-damaging agents [12, 15, 31] and following NRH supplementation to increase in NAD^+^ levels [23]. In our studies, PARGi augmented prexasertib-induced replication stress responses, accumulation of PARylation, increased DNA damage and synergistic OC cell death. These results further support that PARG-mediated efficient and timely dePARylation is essential for cell survival in responses to genotoxic stress. These results provide critical information for clinical use of PARGi for OC and in designing rational drug combinations, particularly to treat chemoresistant OC. Synthetic lethality of CHK1 and PARG inhibitors have already been established [15, 61]. Data from clinical studies suggests ultimate emergence of resistance to PARP-targeted therapies. Compelling synergy data of CHK1i and PARGi combination in these studies suggest potential therapeutic regimen to treat ovarian tumors that develop resistance to platinum and PARP inhibiting drugs.

Cellular levels of NAD^+^ are altered in cancer cells due to oncogenic and metabolic stress, and decreased levels of NAD^+^ compromises cells’ ability to efficiently repair DNA lesions [22, 23, 65, 66]. As speculated, we observed significant depletion of NAD^+^ in OC cell lines treated with prexasertib. This indicates DNA damage induced PARylation of proteins by PARP1 expend cellular NAD^+^, which in turn causes metabolic stress [67]. Further inhibition of PARG in these conditions prevents hydrolysis of PAR and might create metabolic stress in these cells as indicated by NAD^+^ depletion. Additionally, NAD^+^ is also a cofactor for cellular ALDH superfamily of enzymes [24]. Particularly, ALDH1A1 is upregulated in OC and a major biomarker for ovarian cancer CSCs [55, 68]. Interestingly, transient inhibition of PARG causes depletion of cellular NAD^+^ levels and decreased ALDH activity as indicated by AldeFlour assays. Together these observations indicate that PARG inhibition could overcome chemoresistance in OC by suppressing cancer cell stemness properties. Consistently, our results show a greater synergy of CHK1i and PARGi combination in isogenic chemoresistant OC cells compared to parent chemosensitive cells. Additionally, PARGi alone caused increased activation of CHK1 as indicated by pCHK1(S296) compared to their chemo-naive OC cells, suggesting an important role for PARG and CHK1 in chemoresistance. Similarly, chemoresistant cells also showed increased DNA damage marker pH2AX(S139) compared to their sensitive counterparts. An important readout for CSCs is their ability to form tumor spheres in 3D growth assays and tumor organoid cultures. The combination of CHK1i and PARGi also significantly attenuated primary OC patient-derived cell’s ability to form tumor organoids.

Collectively, results from this study demonstrated that combination of drugs targeting CHK1 and PARG in OC cells not only causes persistent heavy PARylation and DNA damage, but also abrogates cell cycle checkpoints, depletes cellular NAD^+^ levels, and inhibits DNA repair, causing cancer cells to undergo mitotic catastrophe, as well as synergistic lethality in OC cells (Fig. 8F). Additionally, depleted NAD^+^ levels compromise CSC maintenance by inhibiting ALDH activity, an important signal in OC. Even though numerous PARG inhibitors are proposed as a therapeutic indication in multiple cancers [69, 70], the potent PARGi that show in vivo antitumor activity is yet to be established, which limited our preclinical study in the animal models. Nevertheless, our studies provide novel mechanistic insights into the therapeutic potential of this combination and provide preclinical evidence to further develop this combination therapy in treating chemoresistant OC.

## Materials and methods

### Cell lines, culture method, and reagents

Human ovarian cancer cell lines OVCAR8, SKOV3, OVSAHO, and A2780 were purchased from ATCC, Manassas, VA. All four cell lines were cultured in the Dulbecco’s modified Eagle medium (Corning, Manassas, VA), supplemented with 10% fetal bovine serum (Omega Scientific Inc., Tarzana, CA) and 1% penicillin–streptomycin (50 U/mL, 50 μg/mL, Invitrogen, Eugene, OR). Prexasertib (Selleckchem, Houston, TX), PARG inhibitor- *PDD00017273*, abbreviated as PDD, (Selleckchem, Houston, TX), were dissolved in DMSO and used at the specified concentrations and times as indicated. The following primary antibodies were used for western blotting: PAR (Santa Cruz Biotechnology, Santa Cruz, CA), pH2AX(S139) (Millipore, Billerica, MA), pCHK1(S296) (Cell Signaling, Danvers, MA), pCHK1(S317) (Cell Signaling, Danvers, MA), pCHK1(S345) (Cell Signaling, Danvers, MA), CHK1 (Santa Cruz Biotechnology, Santa Cruz, CA), pRPA32(S33) (Cell Signaling, Danvers, MA), ALDH1A1 (Cell Signaling, Danvers, MA) and GAPDH (Santa Cruz Biotechnology, Santa Cruz, CA).

### Presto blue cytotoxicity assay/Bliss synergy assay

The Presto Blue^TM^ cell viability assay (Invitrogen, Carlsbad, USA) was performed to examine the cytotoxicity of prexasertib, PDD and their combination treatment in OVCAR8, OVSAHO, SKOV3, A2780 and A2780/CP70 (isogenic platinum drug-resistant cell line) cell lines. Approximately 5000 cells/well were seeded and incubated in a CO_2_ incubator at 37 °C for overnight and were exposed to different concentrations of prexasertib, PDD and their combination for 72 h. The cells were incubated with media containing 10% Presto Blue^TM^ reagent for additional 3–4 h and absorbance was measured at 570 nm using a microplate reader (Biotech Instruments, USA). The data were further processed to obtain Bliss synergy score using Microsoft Excel and GraphPad prism (9.1.0). Each experiment was repeated thrice keeping all conditions constant.

### Protein expression by western blot

Cells were placed on ice and washed twice with ice-cold PBS, and cell lysates were collected using either cytoskeletal (CSK) buffer as described previously [71, 72], (10 mM PIPES at pH 6.8, 100 mM NaCl, 300 mM sucrose, 3 mM MgCl_2_, 1 mM EGTA, 0.1 mM ATP, 0.1% Triton X-100 freshly supplemented with 1 mM dithiothreitol, 1× protease and phosphatase inhibitors with EDTA) or RIPA buffer (50 mM Tris-HCl at pH 7.4, 150 mM NaCl, 1% Triton X-100 or NP-40, 0.5% Sodium deoxycholate, 0.1% SDS, 1 mM EDTA, 10 mM Naf with freshly supplemented with 1× protease and phosphatase inhibitors). Either Bradford reagent for CSK, or DC reagent for RIPA were used to estimate protein content, and the proteins were equilibrated using either CSK buffer or ddH_2_O with 6× Laemmli buffer and heated at 100 °C for 15 min. The proteins were resolved on gradient polyacrylamide gels and then transferred onto nitrocellulose membrane using BioRad Trans-Blot Turbo system. The membranes were blocked using 2.5% blocking grade blocker (BioRad, USA) in 1× Tris-buffered saline in 0.1% Tween 20 (TBST) and incubated with the primary antibody overnight on a rocking platform at 4 °C. Membranes were then washed three times with 1× TBST, and secondary antibody was added and incubated further for an hour at room temperature. The membranes were again washed three times with 1× TBST and exposed to Western lightning plus ECL (Perklin Elmer, USA) and developed in a dark room with Konica Minolta equipment.

### Immunofluorescence

SKOV3 cells were seeded into the 35 mm glass-bottom dishes and incubated overnight for adherence. Cells were then treated with DMSO, 5 nM prexasertib, 5 µM PDD and combination drugs for 24 h. Cells were washed with ice-cold PBS, fixed with 0.5% formaldehyde for 10 min and quenched with 0.1 M glycine/TBS for 5 min. Then, cells were washed and again fixed in 100% methanol ( −20 °C) for 5–10 min at room temperature. The cells were washed and blocked in 10% goat serum for 45 min at room temperature followed by three washes with PBS. Three hundred microliters of primary antibodies [pH2AX(S139) (Cat No: 05636, Millipore), pRPA32(S33) (Cat No: 10148S, Cell Signaling), PAR (Cat No: 4335MC100, Trivegen)] in 1:1000, 1:200 and 1:200 ratio, respectively in PBS were added to each dish containing cells and were incubated overnight at 4 °C in a humidified chamber. After overnight incubation, the dishes were washed three times with PBS and incubated with (200 µL/dishes) fluorescence tagged secondary antibodies (Molecular Probes) for 2 h at room temperature and mounted with Vectashield containing DAPI (Cat No: H-1500, Vector). Images were taken using Nikon T1-E with A1 Confocal microscope and analyzed with ImageJ.

### Cell cycle analysis

OVCAR8 and SKOV3 cells were grown in the 10 cm culture plates at (50–60) % confluency. The cells in their respective plates were treated with DMSO, prexasertib and PDD either as single agents or their combination for 24 and/or 48 h. After drug treatment, cells were trypsinized and washed with ice-cold PBS and processed for flow cytometry as described previously [73]. Briefly, cells were then re-suspended in ice-cold ethanol and fixed by incubating overnight at −20 °C. After incubation, cells were washed with PBS, stained with propidium iodide (PI) (Invitrogen, Eugene, OR), and analyzed for cell cycle profile by flow cytometry using a BD Accuri (BD Biosciences) flow cytometer. ModFit LT 5.0 and/or FlowJo_V10 software were used to calculate the percentage G_0_/G_1_, S, and G_2_/M phases and were averaged from three independent experiments.

### Nuclear distortion assay

To observe distorted nuclei-related morphological changes associated with mitotic catastrophe, OC cells (SKOV3 and OVCAR8) were seeded into the 35 mm glass-bottom dishes and incubated overnight for adherence. Cells were then treated with DMSO, 5 nM prexasertib, 5 µM PDD and prexasertib + PDD combination drugs for 24 h. The treated cells were fixed with ice-cold methanol for 5 min, on ice. The cells were then washed thrice with PBS and stained with DAPI (Invitrogen, Eugene, OR) for 5 min and incubated overnight at room temperature. Then the cell plates were imaged using Nikon T1-E with A1 Confocal microscope.

### Comet-Chip assay

Cell size was measured using the Invitrogen Countess 3 automated cell counter (ThermoFisher Scientific; Waltman, MA) to ensure the appropriate size of cells are selected for the Comet-Chip microwells. The 30-micron-sized Comet-Chip (Trevigen, Gaithersburg, MD) used for this experiment contains 96 wells and approximately 500 microwells. Comet-Chip assay was performed under alkaline conditions using the Comet Assay Kit as per the manufacturer’s instructions and as reported previously [74]. In brief, OC cells were treated 24 h with DMSO, 5 nM prexasertib, and 5 μM PDD and their combinations. Pre-treated cells were harvested, and gravity loaded into the microwells for 30 min in the Comet-Chip calibrated previously at room temperature. The chip was then washed multiple times with PBS and sealed with 1% low melting point agarose at a ratio of 1:10 (v/v). Comet-Chip was immersed in a lysis solution for 30 min at 4 °C. The chip was electrophoresed (22 V for 50 min at 4 °C) in a horizontal electrophoresis apparatus that contains alkaline solution (200 nM NaOH, 1 mM EDTA, 0.1% Triton X-100). After electrophoresis, the chip was then neutralized using 0.4 M and 20 mM Tris-HCl pH 7.4. The chip was then stained with SYBR gold and destained with 20 mM Tris-HCl pH 7.4 to visualize cellular DNA using Zeiss Axio fluorescence microscope. Fluorescence images were analyzed using the ImageJ comet program to demarcate the “head” and “tail” regions of each comet. The comet tail area was measured, and calculations were averaged from three independent experiments.

### Clonogenic survival assay

The colony formation assays were used to investigate the cytotoxic effect of drugs and how it affects cell survival, proliferation, and colony formation. Five thousand cells per well for high-density assay and 500 cells per well for low-density assay were seeded into 6-well culture plates and incubated overnight for adherence. Cells were then treated with DMSO or various concentrations of prexasertib and/or PDD and cultured for colony formation over a period of 5–7 days. After colony formation, growth medium was removed, cells were washed with ice-cold PBS thrice, and then fixed in ice-cold methanol for 5 min. Methanol was replaced with 1% w/v crystal violet (Invitrogen, Eugene, OR) for staining, and after 10 min, the wells were washed under gentle tap water, and plates were allowed to dry at room temperature. In case of high-density assay, colonies were imaged and the colony intensity was measured using ImageJ software, as described previously [75]. The colony intensity calculations were averaged from three independent experiments. However, in case of low-density assay, colonies were imaged and counted manually. The total number of colonies were counted and averaged from three independent experiments.

### NAD^+^ assay

EnzyFluo NAD^+^/NADH Assay Kit was purchased from BioAssay Systems and total NAD^+^ was measured as per the manufacturer instructions. About 500 cells of OVCAR8 cells were seeded in the 60 mm plates and incubated overnight for adherence. The cells were monitored continuously until at least >1,000,000 cells could be predicted in each plate. Then the cells were treated with 1 µM of prexasertib for 0, 2, 4, and 8 h. On the other side, OVCAR8 cells were treated with DMSO, 1 µM of prexasertib, 5 µM of PDD and combination drugs for 8 h. After every time point treatment in each of the above cases, cells were lysed and homogenized in NAD extraction buffer and the lysates for each time point were collected from their respective plate. For sample normalization, a BCA assay was performed. The standard curve for NAD was made by serial dilution as instructed, and 50 µl standard or cell lysate were used in a working solution. Greiner CELLSTAR 96-well flat black plates were used, and fluorescence intensity reading was taken at λ_ex/em_ = 530/585 nm. Total NAD in samples was calculated based on the standard curve. The experiments were performed in triplicates.

### siRNA transfection

The siRNAs (siControl and siCHK1) used in this study were purchased from Dharmacon (Lafayette, CO). Sequences for siControl and siCHK1 are 5′-UAG CGA CUA AAC ACA AUU-3′ and 5′-GCG UGC CGU AGA CUG UCC AUU-3′, respectively. siRNAs double transfection was performed each at 24 h interval using Lipofectamine® RNAiMAX Reagent (Invitrogen, CA) based on the protocol supplied by the manufacturer.

### Survival assay

The cell survival assay was performed by using Presto Blue™ reagent purchased from (Invitrogen, Carlsbad, USA) to quantify the survival percentage of siCtrl and siCHK1 transfected cells (OVCAR8 and SKOV3) with and without PDD treatment. Approximately, 5000 cells/well from each group were seeded and incubated in a CO_2_ incubator at 37 °C for overnight and were exposed to different concentrations (0, 0.5, 1, 2.5, 5, 10, 20, and 40 µM) of PDD for 72 h. The cells were incubated with media containing 10% Presto Blue™ reagent for additional 3–4 h and absorbance was measured at 570 nm using a microplate reader (Biotech Instruments, USA). The data were further processed to obtain a survival graph using Microsoft Excel and GraphPad prism (9.1.0). The experiment was performed in triplicates.

### AldeFluor assay

ALDEFLUOR^TM^ Assay kit was purchased from STEMCELL^TM^ Technologies (Catalog #01700). For the detection of aldehyde dehydrogenase (ALDH) enzymatic activity, about 1 × 10^6^ of each SKOV3 and OVCAR8 cells were placed in ALDEFLUOR buffer and processed for staining with the ALDEFLUOR kit according to the manufacturer protocol. Briefly, single cells were suspended in a fluorescent activated ALDEFLOUR^TM^ Reagent (BAAA) diluted with ALDH Assay buffer. The specific ALDH inhibitor Diethylamino benzaldehyde (DEAB) was used as a negative control at a concentration of 50 mmol/L in each of this experiment. The ALDH activity upon treatment with PARGi (PDD) was performed using BD Accuri (BD Biosciences) flow cytometer. FlowJo_V10 software was used to calculate the ALDH +ve cells in each group averaged from three independent experiments.

### Primary OC cells 3D organoid assay

Three different primary OC cells (TX-OV-076, TX-OV-186, and TX-OV-285) were received from TTUHSC cancer center. The cells were revived and grown initially in the 10 cm culture plates. After sufficient confluency, the cells were trypsinized, dispersed into single cells and approximately 10,000 cells were seeded per well in triplicates in the ultra-low attachment six-well plates (Cat No: 3471, Corning) and cultured in media [DMEM/F-12 media supplemented with 20% Fetal bovine serum (Cat No: FB-02, Omega), 50X B-27 supplement (Cat No: 3248, Gibco), 100X N-2 supplement (Cat No: 3330, Gibco), (1:1) growth factors supplement {recombinant human epidermal growth factor (EGF) (Cat No: AF-100-15, Peprotech) and fibroblast growth factor (FGF) (Cat No: 100-26, Peprotech)}] to form spheres or organoids. After overnight incubation of cells at 37 °C, they were treated with DMSO, 10 nM of prexasertib, 5 µM PDD and the combination drugs. Cells were continuously monitored for the sphere formation, and fresh media containing all the supplements and drugs were added every 72 h. After the appropriate organoid formation (7–14) days depending upon the individual cell lines, images were captured and analyzed in accordance with their organoid size (length) using in-built ND2 software in the Nikon TE2000 microscope.

### Statistical analysis

One-way ANOVA with Dunnett’s, Games-Howell’s, and Tukey’s multiple comparison test as well as Two-way ANOVA with Dunnett’s, Benferroni’s and Tukey’s multiple comparison test were performed to estimate statistical significance using GraphPad Prism 9.1.0 and Microsoft Excel. Data are presented as mean ± standard deviation and/or standard error of mean wherever applies.

### Supplementary information

Supplementary Figures

Original Western Blots

## Data Availability

All data generated or analyzed during this study are included in this research article and its supplementary information files.
