# Supplementary material for: CHK1 inhibitor induced PARylation by targeting PARG causes excessive replication and metabolic stress and overcomes chemoresistance in ovarian cancer
Source: Cell Death Discov. 2024 Jun 11;10:278. doi: 10.1038/s41420-024-02040-0 (PMC11166985; doi:10.1038/s41420-024-02040-0)
Supplement: Supplementary file 1 — Supplementary Figures [file 41420_2024_2040_MOESM1_ESM.docx]

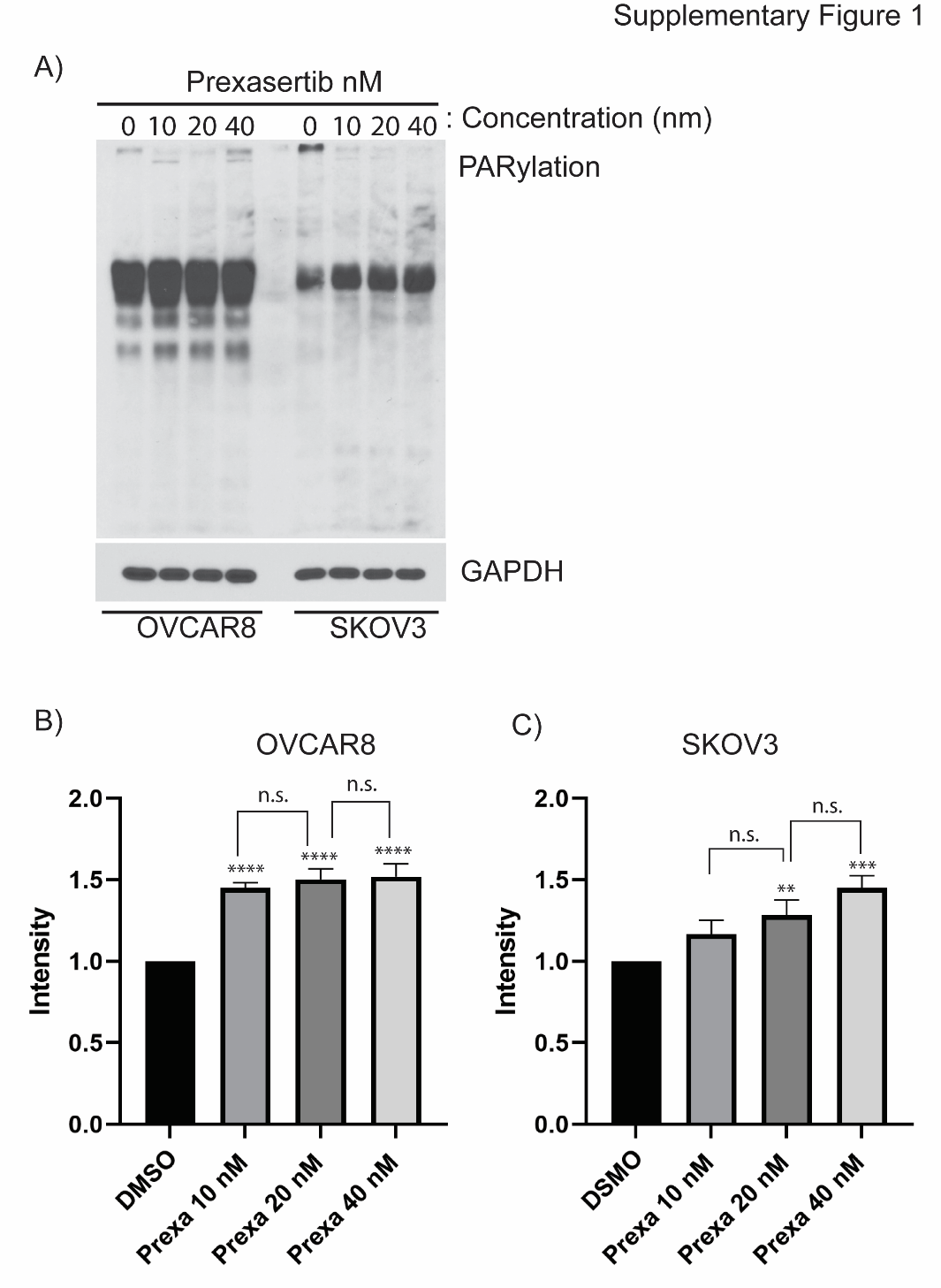


**Supplementary Figure 1: A**) Immunoblot analysis shows PARP-1 mediated PARylation of proteins in OC cells treated with 0 nM, 10 nM, 20 nM and 40 nM of prexasertib in concentration dependent manner. **B, C**) Quantification of elevated levels of PARylated proteins induced by the treatment of 0 nM, 10 nM, 20 nM and 40 nM of prexasertib in OVCAR8 and SKOV3 cells, respectively. ImageJ was used to quantify the intensity of the proteins. All the experiments were repeated three times, and the bar graph denotes their standard deviation. One-way ANOVA using Tukey’s multiple comparison tests were performed to analyze the statistical significance. *n.s.* not significant; ***p*<0.01; ****p* < 0.001; *****p*<0.0001


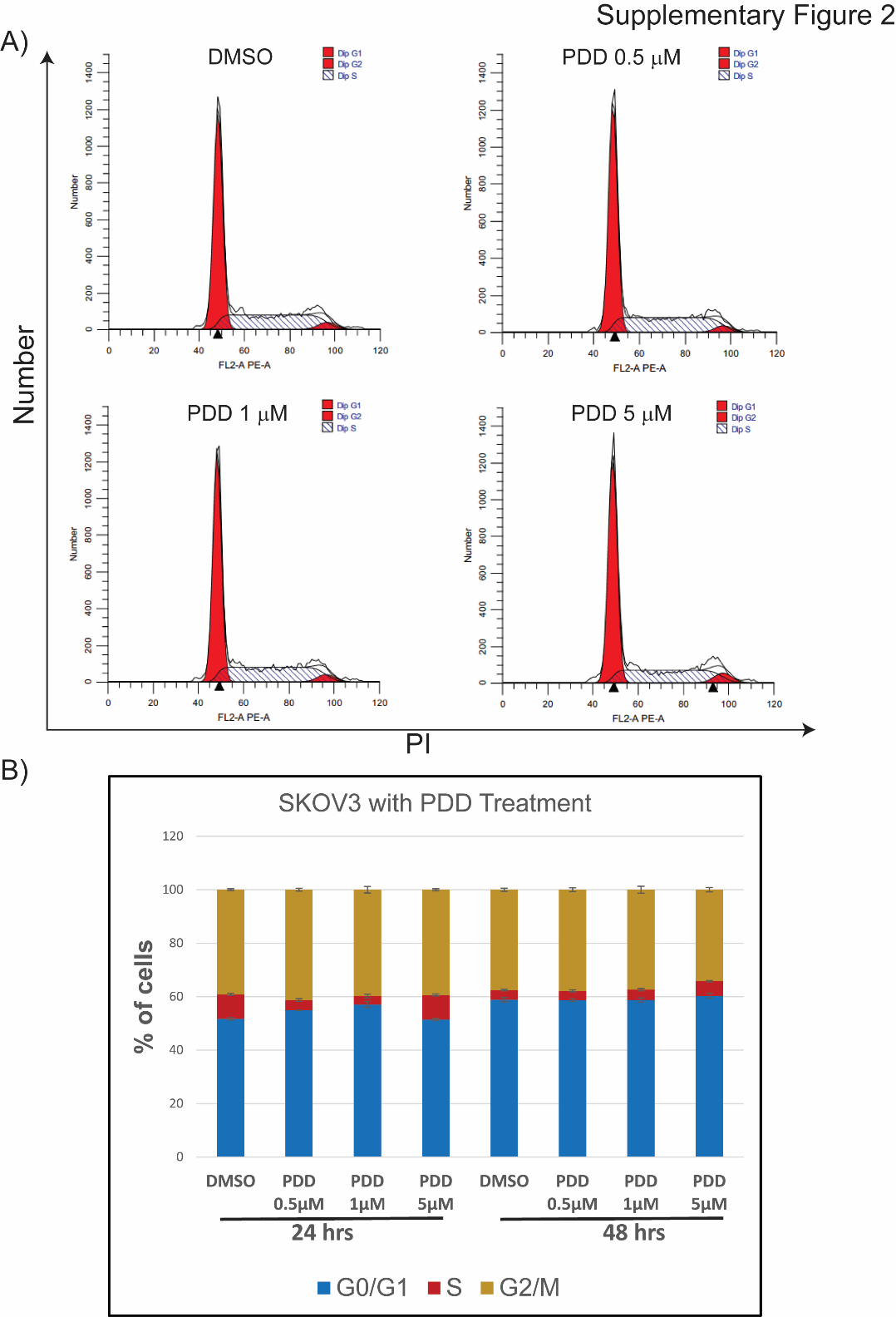


**Supplementary Figure 2: A)** Cell cycle profile of SKOV3 cells treated with DMSO, 0.5 µM, 1 µM and 5 µM of PDD for 24 hours. **B**) Histogram representation of cell cycle profile of SKOV3 cells treated with DMSO, 0.5 µM, 1 µM and 5 µM of PDD for 24 hours and 48 hours. Error bars represent standard deviation from three independent experiments.


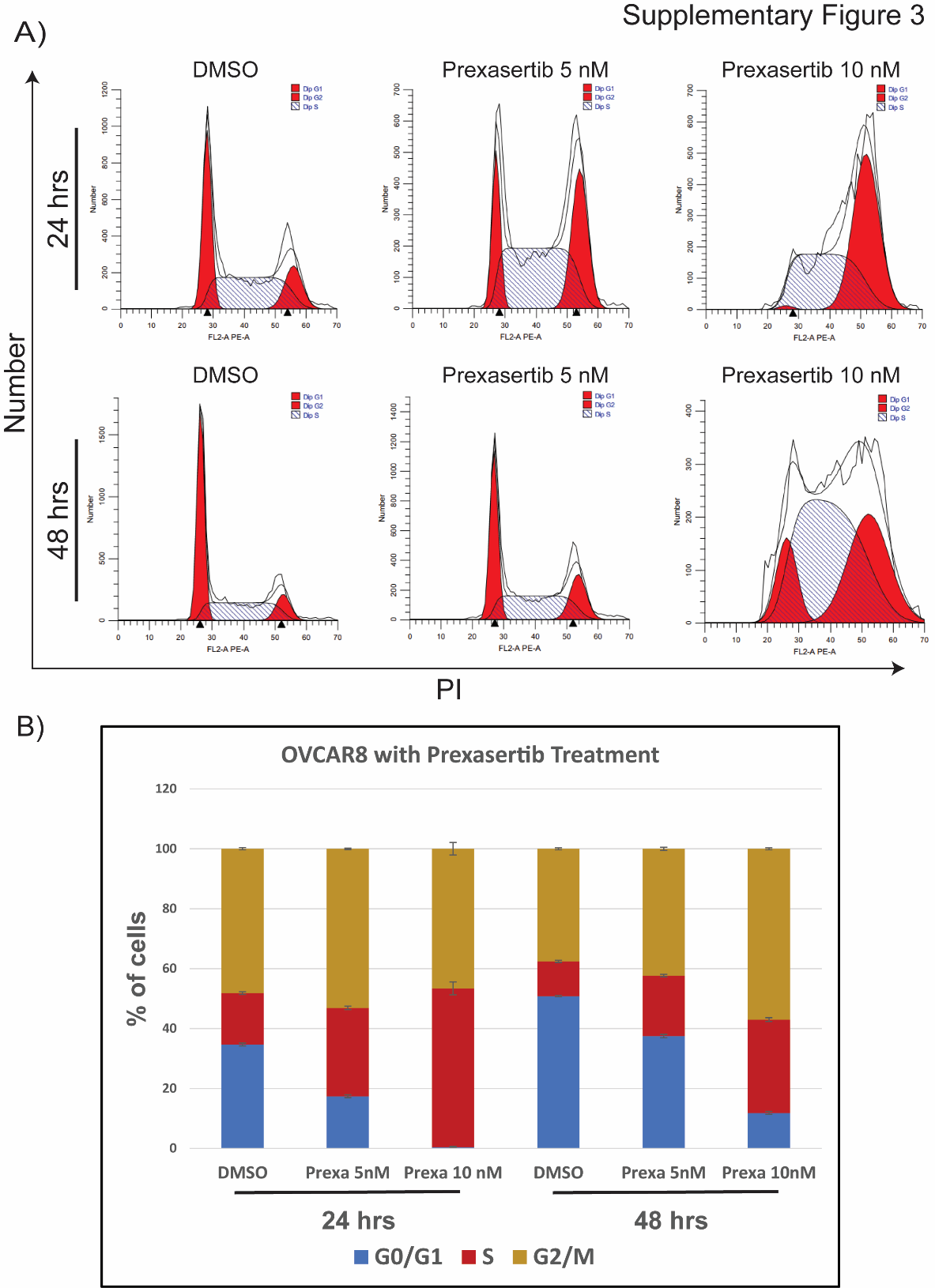


**Supplementary Figure 3:** A) Cell cycle profile of OVCAR8 cells treated with DMSO, 5 nM, and 5 nM of prexasertib for 24 hours and 48 hours. B) Histogram representation of cell cycle profile of OVCAR8 cells treated with DMSO, 5 nM, and 5 nM of prexasertib for 24 hours and 48 hours. Error bars represent standard deviation from three independent experiments.


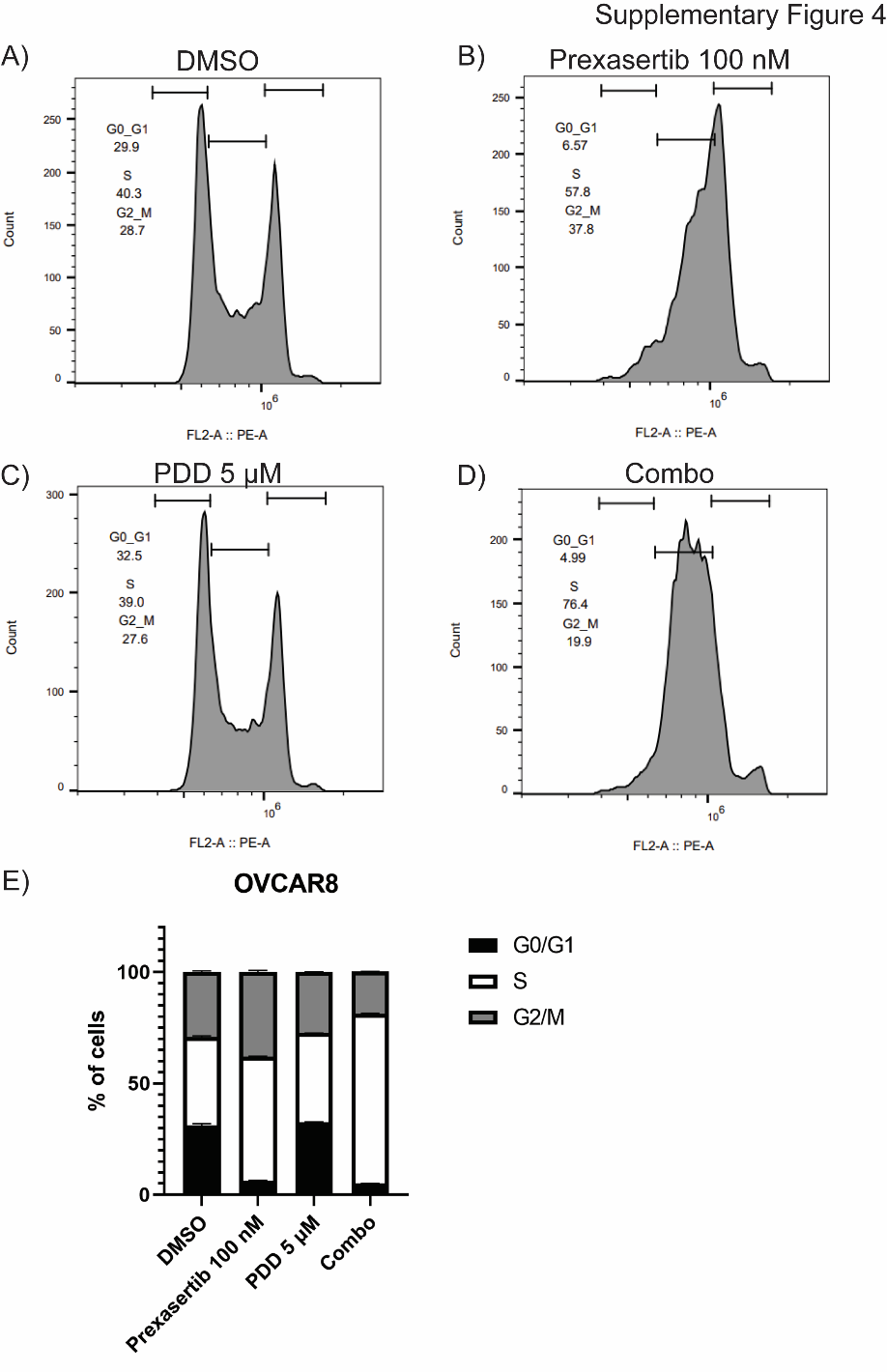


**Supplementary Figure 4:** Cell cycle profile of OVCAR8 cells treated with **A**) DMSO for 24 hours **B**)5 nM prexasertib for 2 hours **C**) 5 µM PDD for 24 hours, and **D**) 5 nM prexasertib for 2 hours plus 5 µM PDD for 24 hours. After 2 hours of prexasertib treatment, cells were washed with PBS thrice and the cells were either treated with medium only or medium containing PDD wherever applies. **E**) Histogram representation of cell cycle profile of OVCAR8 cells treated with DMSO for 24 hours, 5 nM prexasertib for 2 hours, 5 µM PDD for 24 hours, and 5 nM prexasertib for 2 hours plus 5 µM PDD for 24 hours. After 2 hours of prexasertib treatment, cells were washed with PBS three times. Error bars represent standard deviation from three independent experiments.


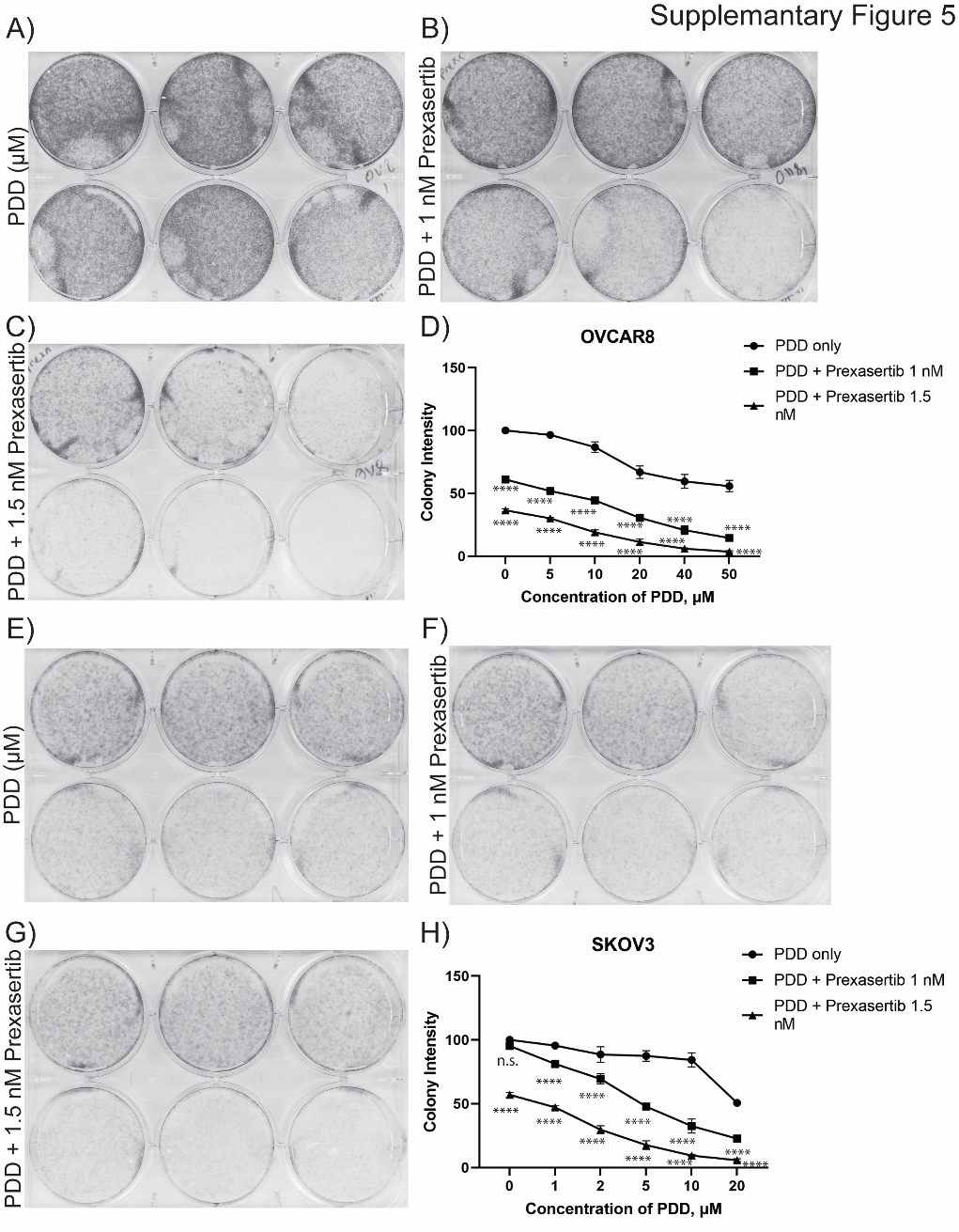


**Supplementary Figure 5: A, E**) Colony assay plates of OC cells with various concentration (0, 5, 10, 20, 40, 50) of PDD (µM) in OVACR8 and SKOV3, respectively. **B, F)** Colony assay plates of OC cells with various concentration (0, 5, 10, 20, 40, 50) of PDD (µM) and or 1 nM of prexasertib in OVCAR8 and SKOV3, respectively. **C, G**) Colony assay plates of OC cells with various concentration (0, 5, 10, 20, 40, 50) of PDD (µM) and or 1.5 nM of prexasertib in OVCAR8 and SKOV3, respectively. **D, H**) Colony intensity graph of OC cells treated with various concentrations of PDD (µM) and/or prexasertib (nM). Error bars represent standard deviation from three independent experiments. Two-way ANOVA with Tukey’s multiple comparison test was performed for statistical analysis. *n.s.* not significant; *****p*<0.0001


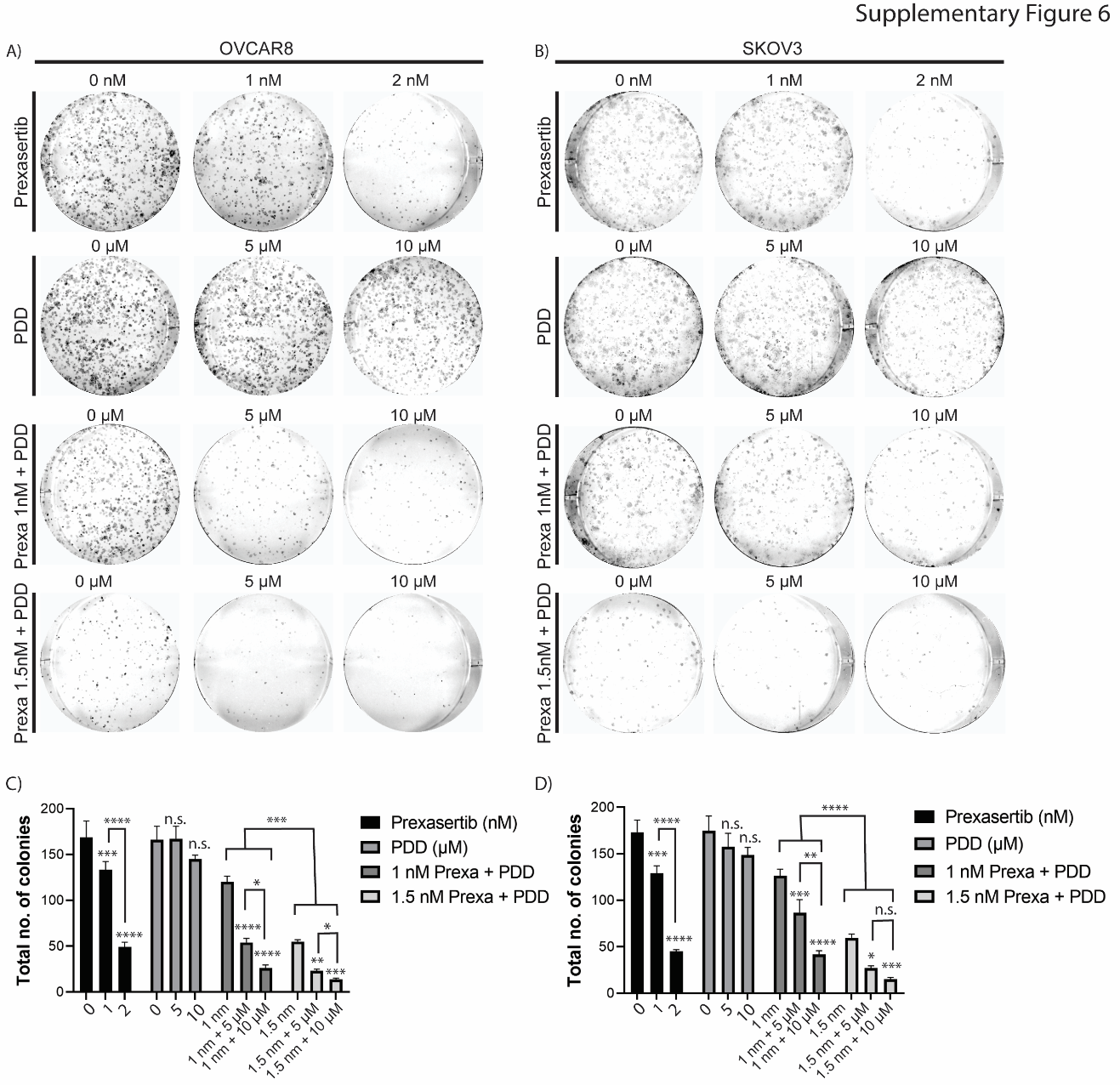
**Supplementary Figure 6: A)** Colony assay plate wells of OVCAR8 cells treated with prexasertib (DMSO, 1 nM, and 2 nM), PDD (DMSO, 5 µM, and 10 µM), prexasertib 1 nM + (0, 5 µM, and 10 µM) of PDD, and prexasertib 1.5 nM + (0, 5 µM, and 10 µM) of PDD. **C)** Histogram representation of total no. of colonies in OVCAR8 cells treated with prexasertib, PDD and their combination as shown in **A**. **B)** Colony assay plate wells of SKOV3 cells treated with prexasertib (DMSO, 1 nM, and 2 nM), PDD (DMSO, 5 µM, and 10 µM), prexasertib 1 nM + (0, 5 µM, and 10 µM) of PDD, and prexasertib 1.5 nM + (0, 5 µM, and 10 µM) of PDD. **D)** Histogram representation of total number of colonies in SKOV3 cells treated with prexasertib, PDD and their combination as shown in **B**. Error bars represent standard deviation from three independent experiments. Two-way ANOVA with Tukey’s multiple comparison test was performed for statistical analysis. *n.s.* not significant; **p*<0.05; ***p* < 0.01; ****p*<0.001; ****p<0.0001
