# Supplementary material for: CHK1 inhibitor induced PARylation by targeting PARG causes excessive replication and metabolic stress and overcomes chemoresistance in ovarian cancer
Source: Cell Death Discov. 2024 Jun 11;10:278. doi: 10.1038/s41420-024-02040-0 (PMC11166985; doi:10.1038/s41420-024-02040-0)

Original PARylation and GAPDH blots for Figure 1 (OVCAR8 cells)

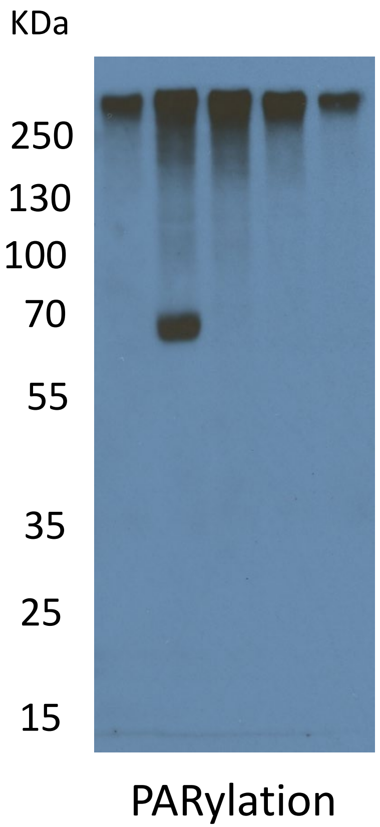

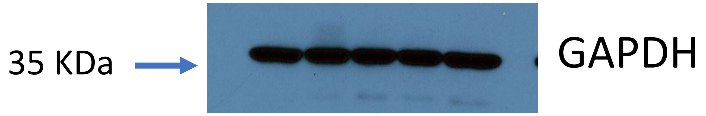

Original PARylation and GAPDH blots for Figure 1 (SKOV3 cells)

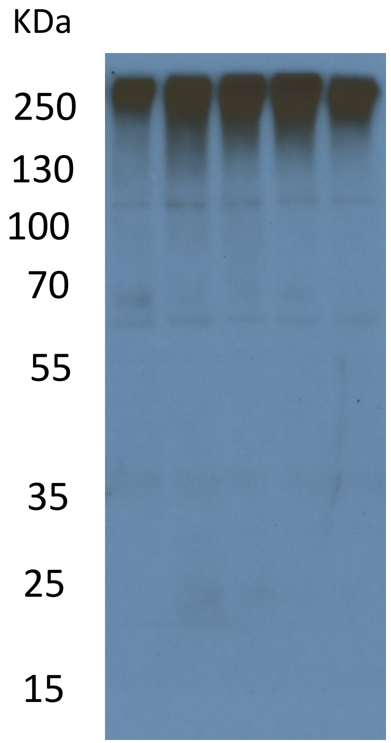

PARylation

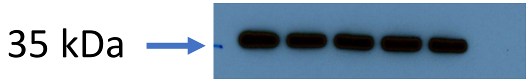

GAPDH

Original blots for phosphorylation of CHK1 and GAPDH for Figure 2A,B
(First column for OVCAR8 and other for SKOV3)

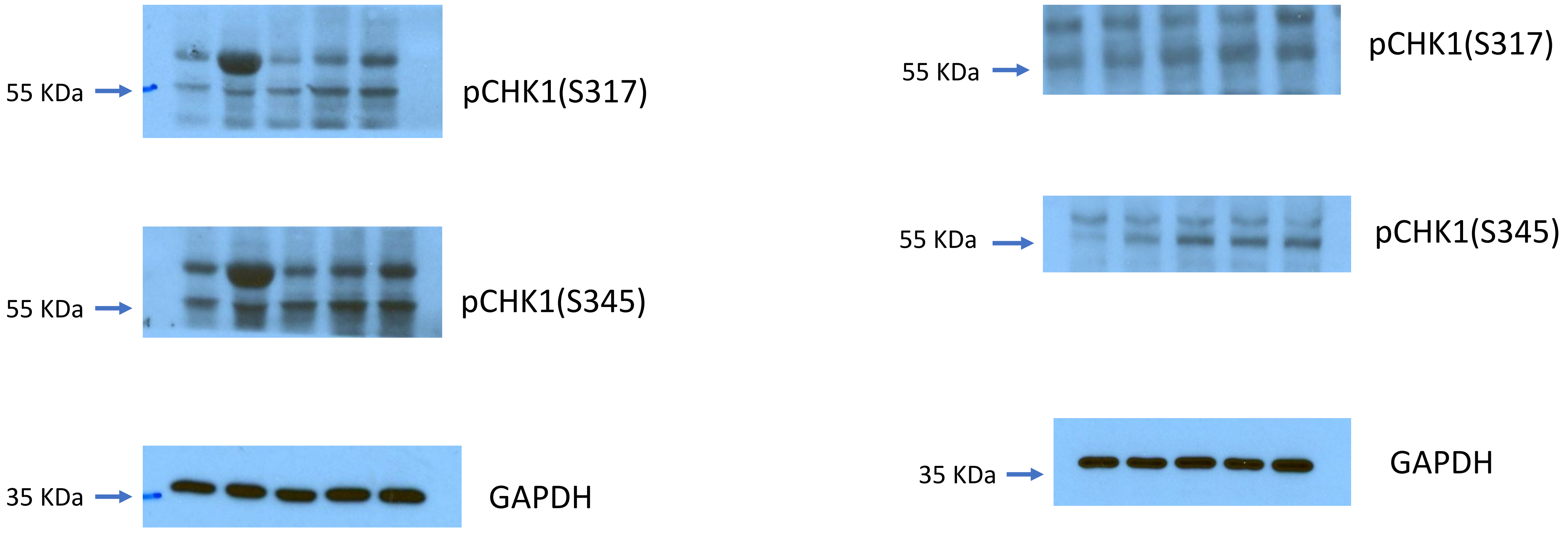

Original PARylation and GAPDH blots for Figure 2C

(First 4 lanes for OVCAR8 and other 4 lanes for SKOV3)

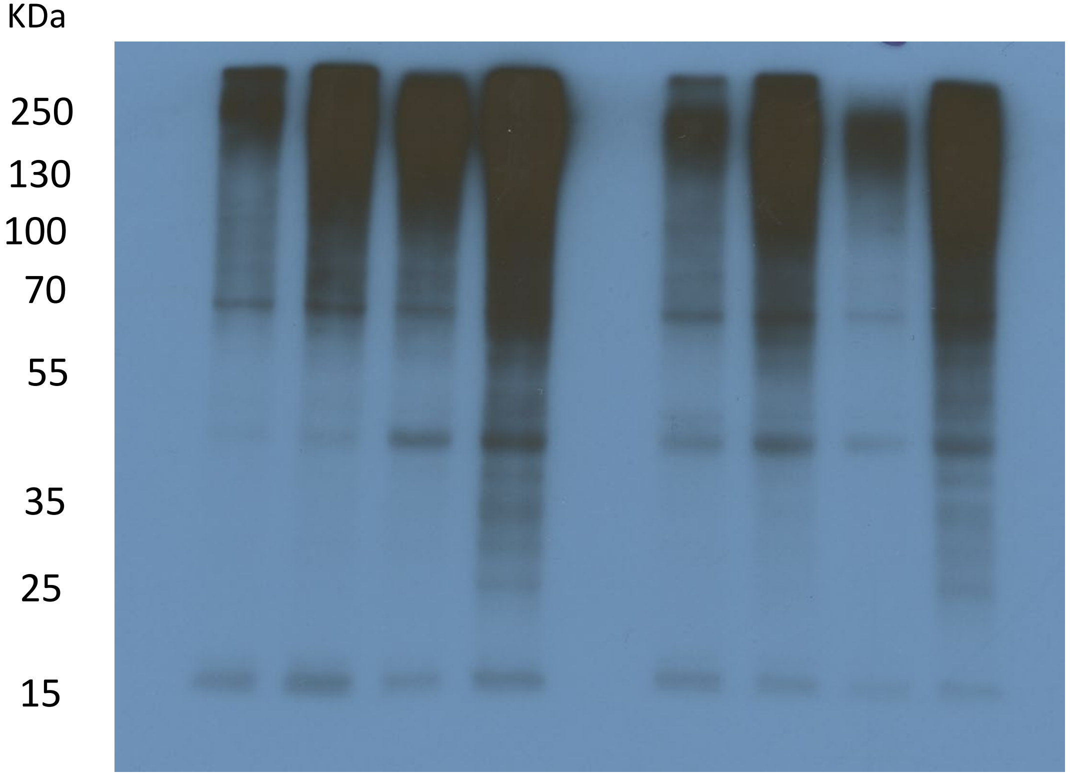

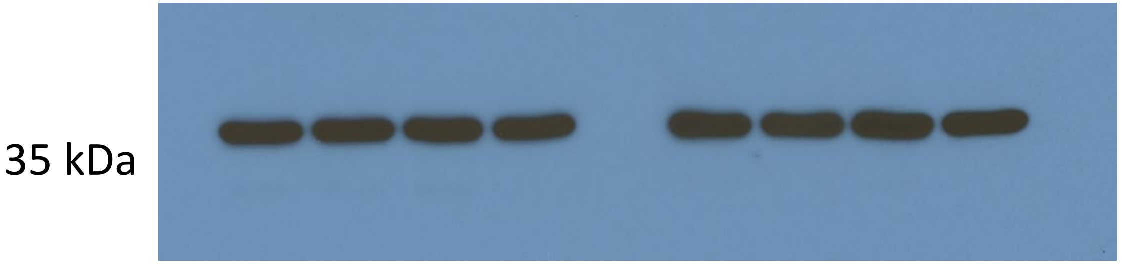

Original blots for phosphorylation of CHK1 and GAPDH for Figure 2D

(First 4 lanes for OVCAR8 and other 4 lanes for SKOV3)

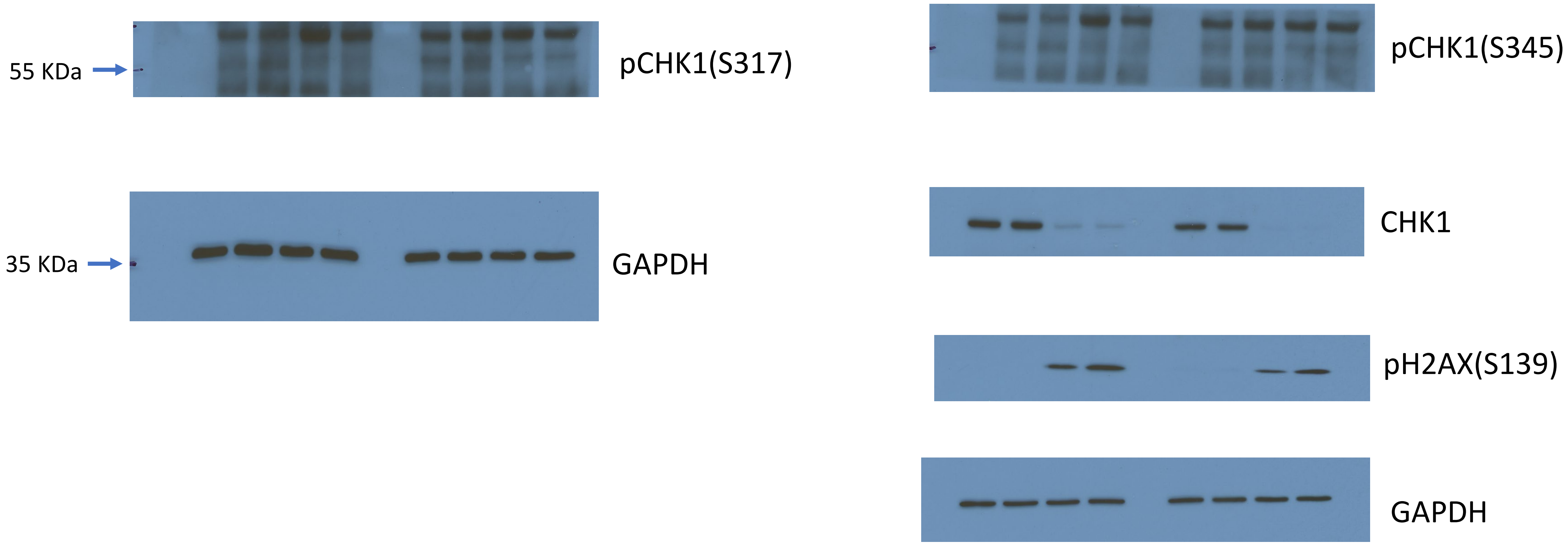

Original PARylation and GAPDH blots for Figure 4A

(First 4 lanes for OVCAR8 and other 4 lanes for SKOV3)

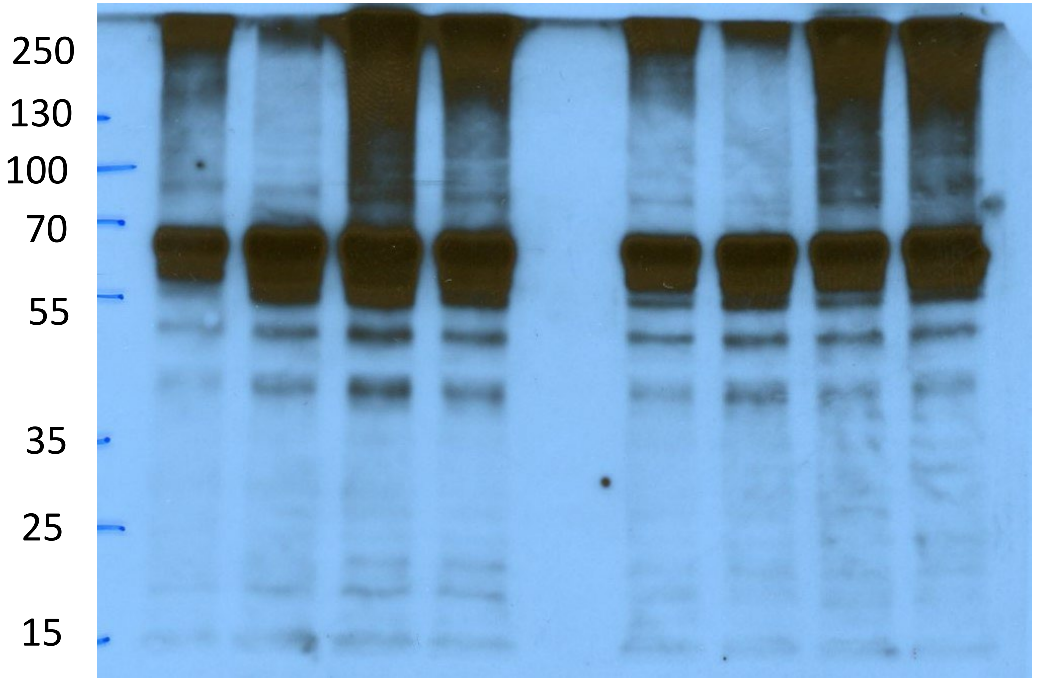

PARylation

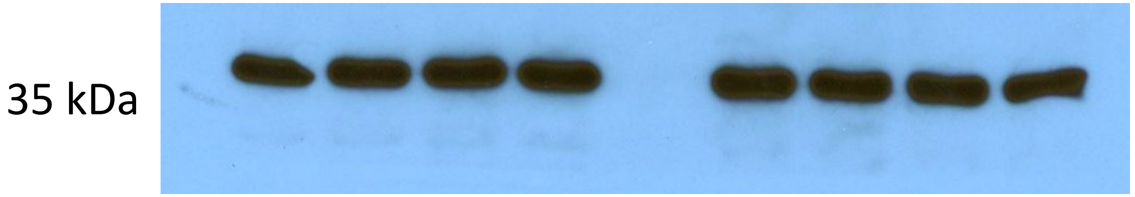

GAPDH

Original blots for pCHK1(S296), CHK1 & pH2AX(S139) for Figure 4A
(First 4 lanes for OVCAR8 and other 4 lanes for SKOV3)

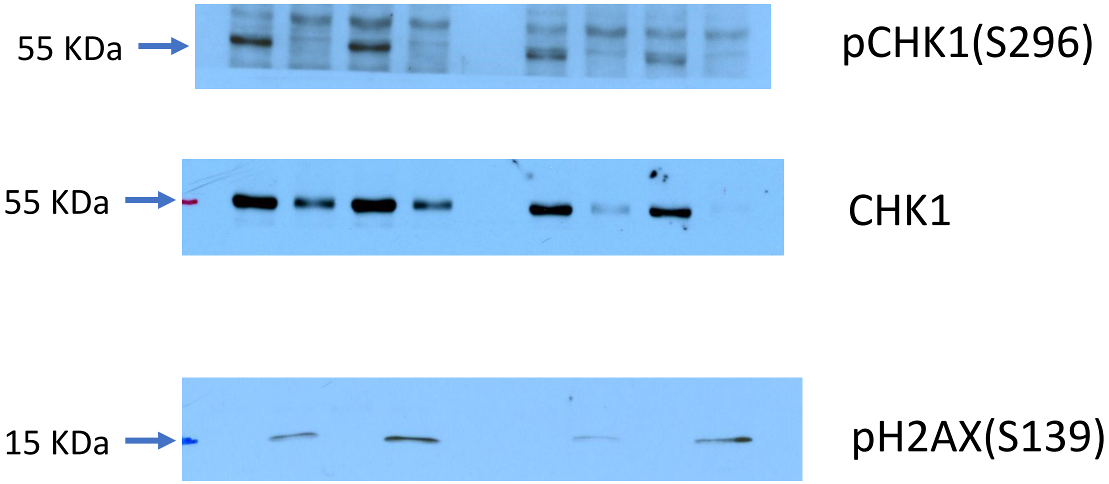

Original blots for pRPA32(S33) for Figure 4B
(SKOV3)

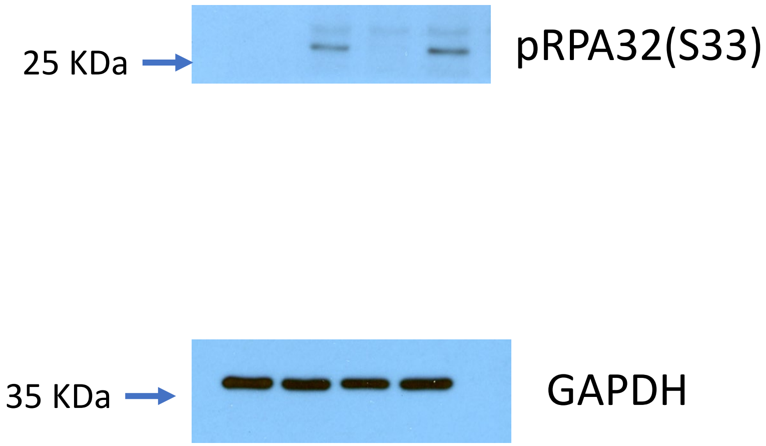

Original blots for pCHK1S296, CHK1, pRPA32(S33), pH2AX(S139) and GAPDH for Figure 8C
(First 4 lanes for A2780 and other 4 lanes for A2780/CP70)

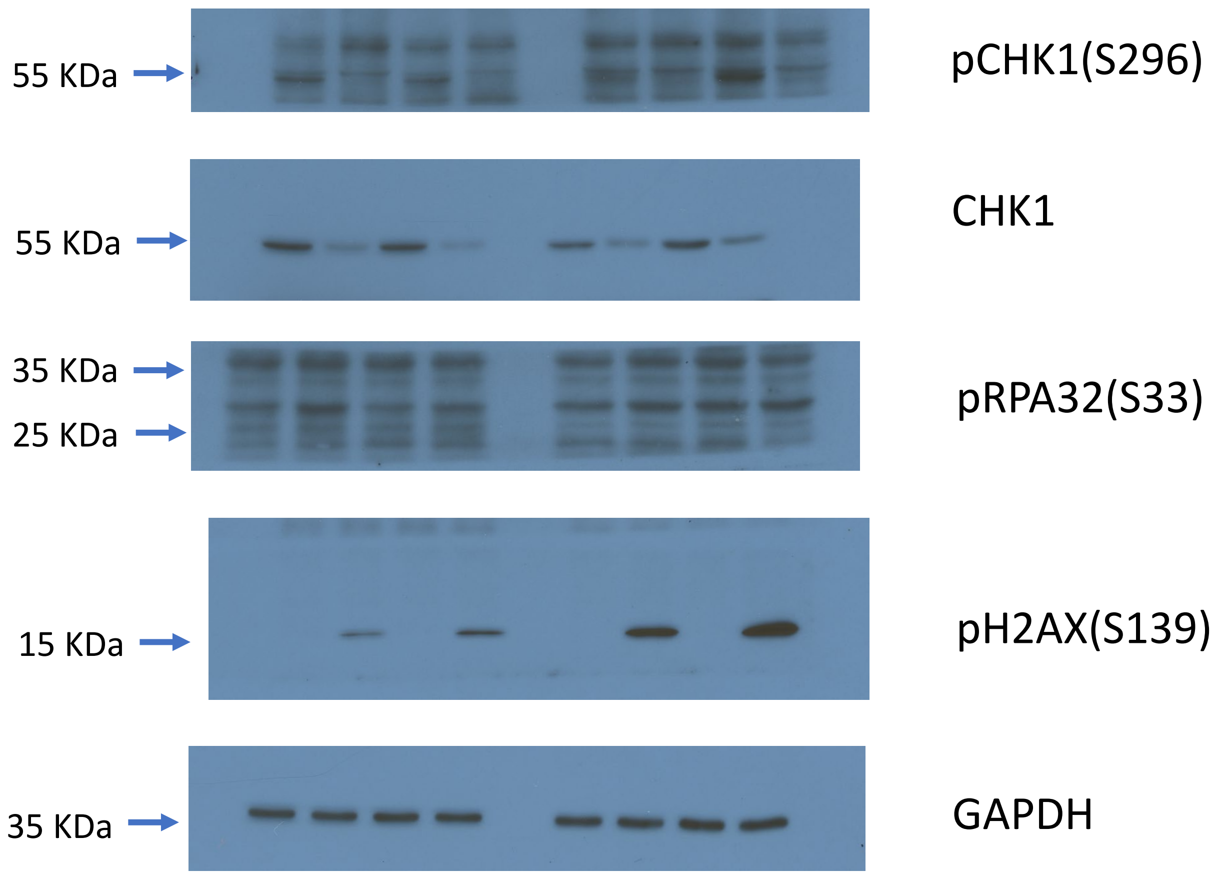

Original blots for ALDH1A1 and GAPDH for Figure 9

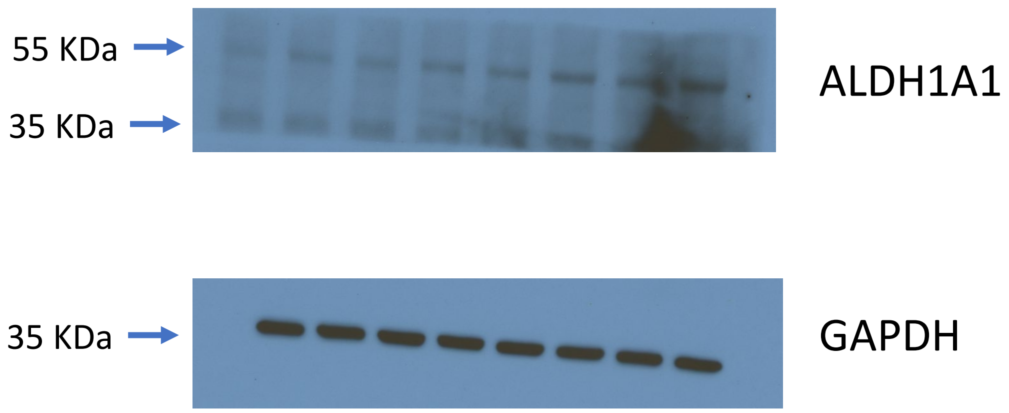

Supplement: Supplementary file 2 — Original Western Blots [file 41420_2024_2040_MOESM2_ESM.pdf]
